# Leveraging genome-wide association analyses with chip and imputed data emerges potential pleiotropic region for four duck growth traits

**DOI:** 10.1038/s41598-025-08852-z

**Published:** 2025-07-02

**Authors:** Eirini Tarsani, Oswald Matika, Kiah McIntosh, Suzanne Desire, Ian C. Dunn, Andrea Talenti, Anne M. Rae, Andreas Kranis, Kellie A. Watson

**Affiliations:** 1 The Roslin Institute and Royal (Dick) School of Veterinary Studies, The University of Edinburgh, Easter Bush, Midlothian, EH25 9RG UK; 2 Department of Animal and Veterinary Sciences, Scotland’s Rural College (SRUC), Easter Bush, Midlothian, EH25 9RG UK; 3 Cherry Valley Farms (UK) Ltd, Blossom Avenue, Humberston, Grimsby, DN36 4TQ England

**Keywords:** Average daily gain, Body weight, Primary feather length, Breast depth, Ducks, Genome-wide association study, Genetic association study, Genome-wide association studies

## Abstract

Average daily gain (ADG), body weight (BW), primary feather length (PRF) and breast depth (BD) are economically important traits in duck production, and understanding the genetic architecture of these traits remains limited. Genome-wide association studies (GWAS) can provide insight into the genetic mechanism underlying these traits. Increasing the power of GWAS by applying approaches such as genotype imputation can improve the ability to detect quantitative trait loci associations with polygenic traits. To increase the power of detecting marker-trait associations in this study, we also exploited imputed data and on a larger sample size. The objective of this study was to investigate marker-trait associations for ADG, BW, PRF and BD in ducks. First, we conducted univariate GWA analyses using chip data (hence medium density data) using 45 K autosomal SNPs and 1445 ducks from single breeding line. Second, we exploited imputed data with a larger sample size (13020) from the same line and performed univariate analyses. Comparison of SNP signals between the medium density and imputed data identified 63 common SNPs that were co-localized on chromosome 4. Several functional candidate genes such as *PPARGC1A*, *LDB2* and *LCORL* were found within or close to the identified region. Indeed, the *LCORL-NCAPG* region has been reported in many mammalian species to be related to growth. Considering results from both analyses, current findings propose novel putative pleiotropic candidate quantitative trait loci (QTL) with the associated genes for the traits we analysed while identifying the most promising QTL region on chromosome 4.

**Supplementary Information:**

The online version contains supplementary material available at 10.1038/s41598-025-08852-z.

## Introduction

Duck (*Anas platyrhynchos*) is the most economically important waterfowl worldwide. Waterfowl production is a rapidly expanding sector, with an increasing global meat duck production from 2.9 million tonnes in 2000 to 6.2 million tonnes in 2021^1^. Globally, the most important duck producing region is Asia with an industry growth as high as 3.5 per cent per year^2^. China is the leading producer with duck meat production jumping from 1.8 million tons in 2000 to 4.8 million tons in 2021^1^. Among various phenotypically diverse indigenous duck breeds, there is the Pekin duck which has undergone intensive artificial selection since Ming Dynasty (A.D. 1368–1644)^3^. Compared with the mallard, Pekin duck shows many striking changes such as white plumage, extraordinary body size and excellent egg production performance^4^. Due to these desirable economic traits, the Pekin duck has become the predominant breed used for meat, feather, and egg production in the global duck industry.

In ducks, several genome-wide association studies (GWAS) have been conducted on quantitative traits such as growth and carcass traits^4–6^, feeding behaviour^6^ and egg production^7^. However, these studies used low numbers of records compared with the current study and in addition there are limited published literature on GWAS for average daily gain (ADG), body weight (BW), primary feather length (PRF) and breast depth (BD) in Pekin ducks.

The current study was setup to investigate the genetic variants affecting growth and carcass traits for ADG, BW, PRF and BD in Pekin nucleus breeding duck flock. Firstly, we conducted univariate GWAS based on genotypes from 60 K SNP array, which we refer to as medium density (MD) SNP on 1445 duck records. Secondly, we increased the sample size to 13,020 records, using imputed genotypes from a parentage SNP array, referred to as low density panel (LD) to increase the power and also verify the if the same QTL observed with the MD would be confirmed on the same traits in the same line.

## Results

### Genotype imputation and imputation accuracy

**Fig. 1:**
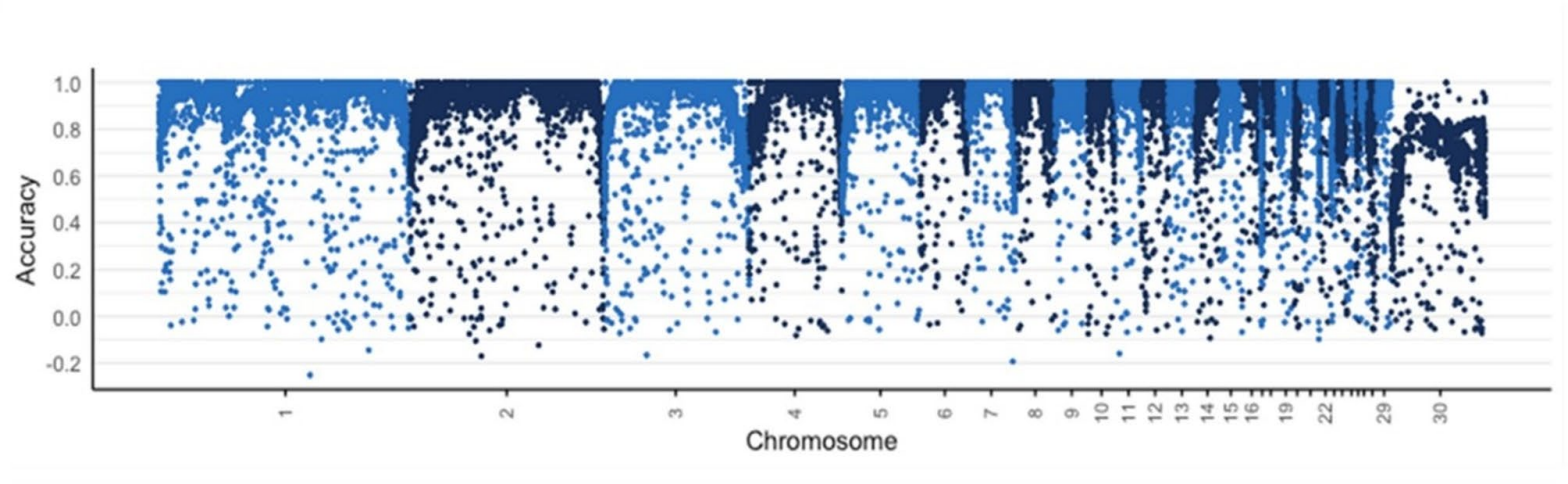
Imputation accuracy on each chromosome.

High imputation accuracies (r^2^) were estimated per chromosome with 0.92 as an average SNP accuracy across all chromosomes. Table 1 shows the 4-fold cross validation findings per group (*n* = 50) while Fig. 1 shows the imputation accuracy (r^2^) estimates per chromosome. We observed poor imputation accuracies at the chromosome ends (see Fig. 1). Our imputation accuracy for chromosome 30, the avian sex (Z) chromosome, was also significantly reduced compared to the autosomes.

**Table 1:** Mean and standard deviation of SNP accuracy in cross-validation groups.

Group of animals	Mean *r*^2^	Standard deviation
1	0.908	0.143
2	0.909	0.144
3	0.912	0.141
4	0.912	0.139

## GWA analyses

### Genetic parameters

We observed heritability estimates ranging from 0.19 (PRF) to 0.54 (BW) from variance component analysis on 1445 MD data estimates based on genomic relationship matrices (Table 2). For the imputed data, the SNP-based heritability estimates ranged from 0.15 (BD) to 0.37 (BW) with consistent results across ASREML and GEMMA (Supplementary Table S1).

**Table 2:** Heritability (diagonal), genetic correlation (below the diagonal), and phenotypic correlation (above the diagonal) estimates for body weight (BW), average daily gain (ADG), muscle depth scan (BD), muscle depth Wit body Wight as a covariate (BDCOV) and primary feather length (PRF) traits using medium density data.

	BW	ADG	BD	BDCOV	PRF
BW	0.53 ± 0.04	0.65 ± 0.02	0.06 ± 0.03	− 0.17 ± 0.03	0.12 ± 0.03
ADG	0.81 ± 0.04	0.31 ± 0.04	0.06 ± 0.03	− 0.11 ± 0.03	− 0.13 ± 0.03
BD	− 0.25 ± 0.10	− 0.11 ± 0.13	0.25 ± 0.04	0.97 ± 0.002	0.04 ± 0.03
BDCOV	− 0.52 ± 0.08	− 0.43 ± 0.11	0.96 ± 0.01	0.31 ± 0.04	0.01 ± 0.03
PRF	− 0.07 ± 0.12	− 0.10 ± 0.14	− 0.02 ± 0.15	− 0.02 ± 0.14	0.19 ± 0.04

We identified the high genetic correlation estimates (r_g_) of 0.96 between BD and BDCOV and 0.81 between BW and ADG from MD genotyped dataset. We also observed moderate to low negative r_g_ of − 0.52, − 0.43, − 0.25, − 0.11, − 0.10, − 0.07, and − 0.02 respectively between BW and BDCOV, ADG and BDCOV, BW and BD, ADG and BW, ADG and PRF, BW and PRF, and BD and PRF (Table 2). The imputed genotyped animals showed similar results with the highest genetic correlation (r_g_= 0.93) observed between BD and BDCOV (Table 3). In addition, we observed positive r_g_ of 0.76, 0.10, 0.05 and 0.03 respectively between BW and ADG, BW and BD, BDCOV and PRF, and BD and PRF. However, negative r_g_ were observed of -0.46, -0.30, -0.28, -0.06 and − 0.06 respectively between ADG and PRF, ADG and BDCOV, BW and BDCOV, BW and PRF, and ADG and BD (Table 3).

**Table 3:** Heritability (diagonal), genetic correlation (below the diagonal) estimates and phenotypic correlation (above the diagonal) between body weight (BW), average daily gain (ADG), muscle depth scan (BD), muscle depth Wit body Wight as a covariate (BDCOV) and primary feather length (PRF) traits using imputed data.

	BW	ADG	BD	BDCOV	PRF
BW	0.39 ± 0.02	0.66 ± 0.01	0.27 ± 0.01	0.13 ± 0.01	0.12 ± 0.01
ADG	0.76 ± 0.02	0.23 ± 0.01	0.18 ± 0.01	− 0.02 ± 0.01	− 0.17 ± 0.01
BD	0.10 ± 0.05	− 0.04 ± 0.06	0.15 ± 0.01	0.97 ± 0.001	0.07 ± 0.01
BDCOV	− 028 ± 0.05	− 0.30 ± 0.05	0.93 ± 0.01	0.31 ± 0.04	0.04 ± 0.01
PRF	− 0.06 ± 0.05	− 0.46 ± 0.05	0.03 ± 0.06	0.05 ± 0.06	0.20 ± 0.01

## Results of QTL identified from univariate analyses using medium density vs. imputed data

**Fig. 2:**
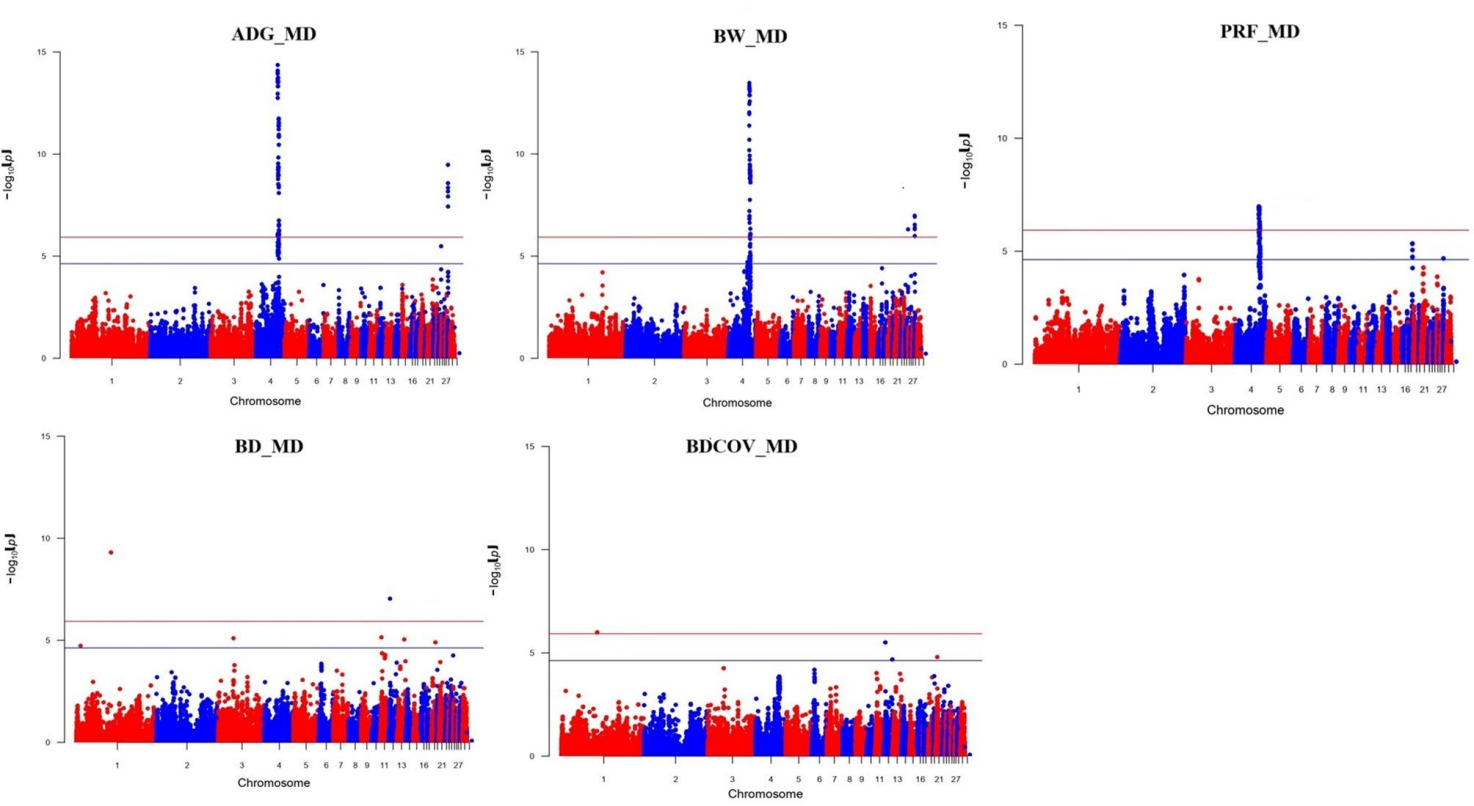
Manhattan plots using medium density data for ADG, BW, PRF, BD and BDCOV. The red horizontal line represents the genome-wide significance threshold, while the blue horizontal line denotes the suggestive significance threshold.

GWAS results obtained from MD genotypes revealed a common QTL region on chromosome 4 for BW, ADG and PRF (Fig. 2). The Q-Q plots along with the genomic inflation factor (λ) estimates from all genomic analyses for MD and imputed data are given in Supplementary Fig. 1. Estimations of the genomic inflation factors were less than 1 (λ = 0.84–0.97) indicating no major concerns to account for population structure in the analysis. We identified 69 SNP located on two chromosomes (4 and 28) that we above the genome-wide significance threshold (*p* < 0.05) for ADG and 65 SNP above the genome-wide significance threshold (*p* < 0.05) for BW located on three autosomes (4, 24, 28, Supplementary Table S2). GWAS results for PRF identified 24 SNPs on chromosome 4 crossing the genome-wide significance threshold level (*p* < 0.05). The same region on chromosome 4 was identified in the three traits (ADG, BW, PRF), however, BD results identified 2 SNP above the genome-wide significance threshold (*p* < 0.05) on chromosomes 1 and 12 (Supplementary Table S2) and BDCOV had 1 SNP crossing the genome-wide significance level on chromosome 1 (Supplementary Table S2). We identified 24 common SNPs for the QTL on chromosome 4 across all three traits for the MD dataset (Supplementary Fig. 2).

**Fig. 3:**
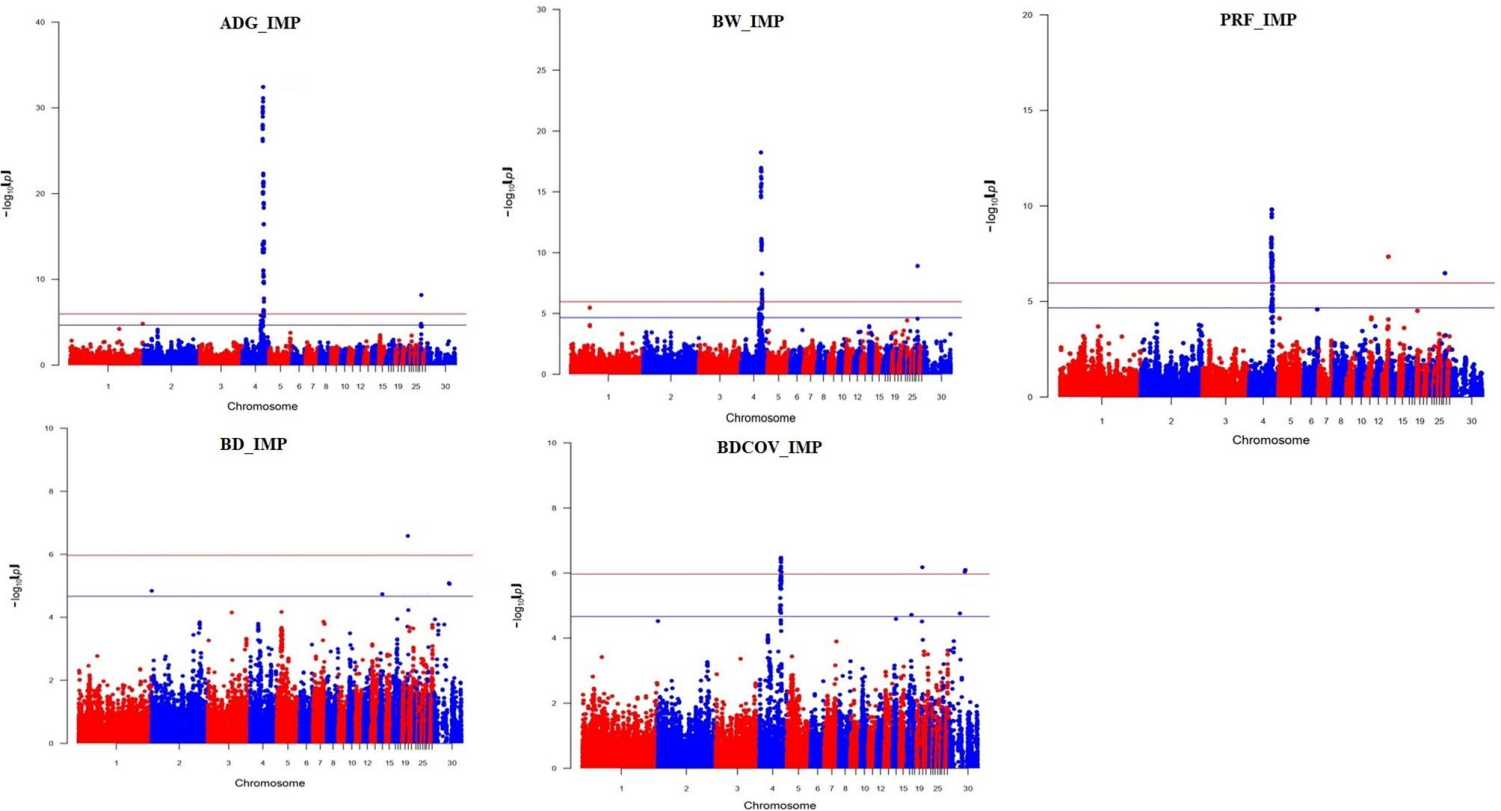
Manhattan plots using imputed data for ADG, BW, PRF, BD and BDCOV. The red horizontal line represents the genome-wide significance threshold, while the blue horizontal line denotes the suggestive significance threshold.

The imputed genotype data Manhattan plots results are given in Fig. 3 and all SNPs above the genome-wide significance threshold (*p* < 0.05) are given in Supplementary Table S3. We identified 83 SNPs above the genome-wide significant level (*p* < 0.05) for ADG (Supplementary Table S3). There were 63 in common SNPs between medium density and imputed data (Supplementary Fig. 3, Supplementary Table S2 and 3). We found 51 SNPs for BW which were above the genome-wide significance threshold (*p* < 0.05) (Supplementary Fig. 3, Supplementary Table S2 and 3) of which 42 were common between medium density and imputed data (Supplementary Fig. 3, Supplementary Table S2 and 3). GWAS results for PRF identified 42 SNP above the genome-wide significance threshold (*p* < 0.05) using imputed data (Supplementary Fig. 3, Supplementary Table S2 and 3) of which 24 SNPs were common between MD and imputed genotypes (Supplementary Fig. 3, Supplementary Table S2 and 3). We found that in BDCOV, we identified the same chromosome 4 QTL as in other traits for BW, ADG and PRF. However, since the chromosome 4 QTL was not identified BD analysis, it was not surprising that no concordance existed between the BD and BDCOV analyses (Supplementary Fig. 3, Supplementary Table S2 and 3). It was reassuring that the BDCOV QTL peak identified on chromosome 4 was the same as that identified for both MD and imputed data for BW, ADG and PRF on the same chromosome making this putative pleiotropic QTL. To ascertain this, we compared the 16 SNPs which crossed the genome wide threshold (*p* < 0.05) with the chromosome 4 GWAS results for ADG using the MD genotypes. We found that 81.25% of the shared BDCOV SNP with ADG were common to both peaks (Supplementary Fig. 4).

The 63 common SNPs identified between the MD and imputed data mapped to the same region on chromosome 4 (57648845–61294508 bp, Supplementary Table S4). In that region, the average LD (r^2^) estimate was 0.82 between the 63 markers (results not shown). The LD heatmap for the top 24 out of the 63 SNPs (chromosome 4) between MD and imputed genotypes is given in Supplementary Fig. 5 that shows the high LD (r^2^ > 0.89) levels between the markers.

## Positional and functional candidate genes

Our search from NCBI database identified 44 putative pleiotropic genes in the 100 kb upstream and downstream from each marker crossing the significant threshold. However, even though the markers were colocalized in the same QTL, they were associated with different traits and genes, for more details see Supplementary Table S4. It was interesting to note that SNPs above the significance threshold were within 16 genes (*DHX15*,* PPARGC1A*,* KCNIP4*,* PACRGL*,* SLIT2*,* LDB2*,* LOC119716756*,* LOC119716899*,* LOC119716898*,* TAPT1*,* PROM1*,* BST1*,* FBXL5*,* CC2D2A*,* C1QTNF7 and CPEB2*) (Supplementary Table S4).

All genes were recognized by ClueGO and used for functional enrichment analysis. The latter analysis revealed a total of 10 enriched GO BPs (Table 4) and 4 participating genes (*FBXL5*,* FGFBP1*,* SOD3* and *TAPT1*). Note that *FGFBP1* and *TAPT1* genes were involved in developmental processes (Table 4).

**Table 4:** Significantly enriched GO biological processes (BPs).

GO ID	GO BP term	*P*-value	FDR *p*-value	Associated genes found
GO:0045743	positive regulation of fibroblast growth factor receptor signalling pathway	0.0006	0.005	*FGFBP1*
GO:0040036	regulation of fibroblast growth factor receptor signalling pathway	0.003	0.009	*FGFBP1*
GO:0090287	regulation of cellular response to growth factor stimulus	0.009	0.01	*FGFBP1*
GO:0055076	transition metal ion homeostasis	0.008	0.01	*FBXL5*
GO:0009791	post-embryonic development	0.003	0.01	*TAPT1*
GO:0006801	superoxide metabolic process	0.003	0.01	*SOD3*
GO:0031146	SCF-dependent proteasomal ubiquitin-dependent protein catabolic process	0.003	0.01	*FBXL5*
GO:0048706	embryonic skeletal system development	0.007	0.01	*TAPT1*
GO:0072593	reactive oxygen species metabolic process	0.006	0.01	*SOD3*
GO:0043161	proteasome-mediated ubiquitin-dependent protein catabolic process	0.02	0.02	*FBXL5*

## Discussion

The present study using relationship matrices from both 60 K MD genotypes and imputed genotypes, demonstrated that growth, carcass and primary feather length traits were heritable. Our genomic heritability estimate for ADG (0.31) was higher than Zhu et al.^8^ (0.15), while our heritability estimate for BW (0.54) was lower than Zhu et al.^8^ (0.64) who also investigated among others the growth traits in Pekin ducks. For BD, our genomic heritability estimate (0.25) was lower than Deng et al.^9^(0.29) who explored breast width in Pekin ducks. For PRF, our heritability estimate (0.19) was higher than Hu et al.^10^ (0.14) who investigated the female feather length in Muscovy ducks, but lower than Hu et al.^10^ (0.37) for the male feather length. In our results, the (co)genetic estimates were slightly, different, this is expected because heritability by their nature are a property of the population and animals sampled from a given the population.

The subsequent GWAS on five growth, carcass and primary feather length traits (ADG, BW, PRF, BD and BDCOV) used 60 K MD genotypes and we investigated the increased power (13 K data records) using imputation genotypes (from a LD parentage to 60 K SNP genotypes) to verify our previous analysis. We hypothesised that increased power had the potential identify novel markers and/or increase the power of detection on those already identified using 1445 MD genotypes. Since the imputation was performed from a LD SNP panel (427 markers) to a 60 K SNP genotypes, given the limited even distribution of SNPs across the genome-wide of the LD panel, our imputation results showed a relatively high imputation accuracy (0.92). Only for chromosome 30, the avian sex (Z) chromosome, the imputation accuracy was reduced compared to the autosomes. This finding aligns with the results reported by Hickey and Kranis^11^ in their study on extending long-range phasing and haplotype library imputation methods to impute genotypes on sex chromosomes, where reduced accuracy on sex chromosomes was observed as a common challenge due to their unique inheritance patterns and structural differences. We also observed lower accuracies at the telomere regions and chromosome ends (Fig. 1) which might have contributed to somewhat lower overall accuracy. We would expect the regions that had lower accuracies might give rise to false-negative tests of association^12^. However, our findings revealed high concordance and higher power resulting in many common SNPs between the GWA analyses using the MD and imputed datasets and higher proportion of SNPs reaching genome-wide significance levels (*p* < 0.05) with lowest *p*-values above 10^− 33^for imputed data as opposed to *p*-value of 10^− 15^ MD genotype for ADG (Supplementary Tables 3 and 4). Specifically, comparison of the genome-wide significant SNPs between the medium density and imputed data resulted in a significant signal peak on chromosome 4 (57648845–61294508 bp). Furthermore, our GWA analyses with the imputed data detected additional marker associations for ADG, PRF and BDCOV traits compared to the GWAS with the medium density data. The imputed genotype data analysis identified the same QTL region on chromosome 4 as in the other traits from both MD and imputed GWAS results.

Our results are in line with those reported in a recent study^13^ on similar traits for shank weight, tibia weight, and femur weight in ducks which identified the same location on chromosome 4 (57066815–57895692 bp and 60606973–61051895 bp) as we found in our study. In addition, our study identified functional candidate genes on chromosome 4 that were also reported by the aforementioned study^13^ for bone quality in ducks, i.e. *LOC113843641 (uncharacterized LOC113843641)*,*CCDC149 (coiled-coil domain containing 149)*,* PPARGC1A (PPARG coactivator 1 alpha)*,* LOC119716899 (uncharacterized LOC119716899)*,* LOC119716756 (uncharacterized LOC119716756)*, *LDB2 (LIM domain binding 2)*,*FGFBP2 (fibroblast growth factor binding protein 2)*,* LOC119716898 (uncharacterized LOC119716898)*,*TAPT1 (transmembrane anterior posterior transformation 1)*,*PROM1 (prominin 1)*,* LOC119716803 (uncharacterized LOC119716803)*,*FGFBP1 (fibroblast growth factor binding protein 1)*,*BST1 (bone marrow stromal cell antigen 1)*,* LOC110352211 (uncharacterized LOC110352211)*, *FBXL5 (F-box and leucine rich repeat protein 5)*,* CC2D2A (coiled-coil and C2 domain containing 2 A) and C1QTNF7 (C1q and TNF related 7).*

From the above functional candidate genes, *PPARGC1A* has previously been reported to play a role in promoting fatty acid oxidation of breast muscle in ducks^14^ while *LDB2* reported as a candidate gene for body size and carcass traits in ducks^5^. *TAPT1* is involved in the development of craniofacial cartilage, which is related to the differentiation of cranial neural crest cells^15^ while *FBXL5* gene has been reported to affect growth in chickens^16^.

The list with the functional candidate genes for the examined traits was not exhausted as *SLIT2 (slit guidance ligand 2) and KCNIP4 (potassium voltage-gated channel interacting protein 4)* genes have already been reported in ducks and/or chickens. *SLIT2* has been reported as candidate gene by a recent GWAS^17^ for plumage colour in ducks. This gene has also been reported to be strongly associated with back yield, feet weight, fat and muscle weight traits in chickens^18^. The *KCNIP4* gene, a member of potassium channel-interacting proteins has extensive physiological functions and insulin secretion was identified as an important QTL region associated with growth traits in chickens^18^.

Although each of these gene’s expression or structure by themselves may have the potential to affect the traits discovered in this study, there is no direct evidence that they do explain the variance observed for ADG, BW, PRF or BDCOV. However, for the locus on chromosome 4 it is adjacent to the *CCKAR* gene that was identified in the chicken as being responsible for a QTL which explained up to 19% difference in growth and body weight and where variation in gene expression was associated with the trait variation observed^19,20^. *CCKAR* expression was lower in animals carrying the high growth allele and they were refractory to the satiating effects of the injection of the receptors ligand, CCK, a known satiety factor released from the intestine and present in the pathways controlling the feeding center in the brain^19^. Indeed, the hypothalamic orexigenic neurons which control feed intake^21^ of the high growth haplotype had a higher expression of their product AGRP.

The *CCKAR* locus in the chicken and duck with their compact genomes is within 2.6 Mb and 2.9 Mb of the LCORL-NCAPG locus *(Ligand dependent nuclear receptor corepressor like (LCORL; ENSGALG00000014421)* and *Condensin complex subunit 3 (NCAPG; ENSGALG00000014425))* in the Chicken GalGal6 2018 and Pekin duck Z2 2020 genome builds respectively. The LCORL-NCAPG locus has been frequently reported to be associated with aspects of stature or weight in many mammalian species^22–32^, . However, a plausible mechanism for the locus effect has proved elusive.

In the chicken and now in the duck, the LCORL-NCAPG region on chromosome 4 has been reported to have pleiotropic effects; including egg quality and weight in laying hens^33,34^, in hybrid laying hens, one strain had a strong association of the locus with body weight^35^the locus was associated with body weight, gizzard weight and intestinal length but not liver weight in a broiler layer cross^26^ and in a F2 population the locus explained variance in bone length and weight^36^oviduct development^37^ and egg size^38^. So, this is a highly replicated observation for the loci across mammalian and avian species.

The possibility that the region around LCORL-NCAPG act as a cis acting long-range enhancer of *CCKAR* such as we have observed for the expression of *SHH* and polydactyly^39^ is a possibility that can be tested for it and other genes such as *SLIT2* in the region. In a study looking at differences in the chicken *CCKAR* promoter it there was a difference in binding sites for *LCORL* between the high and the low growth alleles^40^. This may provide an alternative explanation for the association of variants at the *LCORL* with the growth of chickens although evidence for a cis acting effect exists, at least at a more local level^19^.

In conclusion, our study found that ADG, BW, BD, BDCOV and PRF traits in our duck population were heritable using either MD or imputed genomic relationship matrices. Our GWAS results identified a putative pleiotropic QTL with its associated candidate genes from both MD and imputed genotypes. Results from imputation had more power in regard to previously identified QTL on chromosome 4 for ADG, BW and PRF. In addition, imputed data revealed the same QTL on chromosome 4 for BDCOV irrespective of the fact that MD analysis did not identify this QTL on chromosome 4. Follow-up studies, such as transcription-wide association study, are warranted to elucidate the causative gene(s) on this region.

## Methods

### Ethics statement

No animal experimentation was carried out for this study. This is a purely data analysis study of genotypic and phenotypic records. Data used for analysis were produced by Cherry Valley Farms (UK) Ltd company for their routine animal husbandry and breeding programmes. No data from culled birds was used for the present study. To operate in the animal husbandry Industry, Cherry Valley Farms (UK) Ltd is registered with the Animal & Plant Health Agency (APHA) and has an official veterinarian visit the farms once a month. Additionally, the company is part of the Poultry Health Scheme and the registration number for the farm from which the data were collected is 24/726/014.

### Genotype imputation

For the duck line explored in this study, 18 K individuals were genotyped with a low-density (LD), parentage SNP panel (*n* = 427 SNPs), and 1536 individuals were genotyped with a medium density (MD) 60 K SNP panel to be used as the training set for imputation. The animals for MD were genotyped as a reference population for imputation and genomic selection. The SNP array was custom made at the Roslin Institute for Cherry Valley by the same team that constructed the 600 K SNP array^41^ for chickens.

Using AlphaImpute2 ^42^, a combination of pedigree and population imputation was used to impute medium density genotypes for all individuals. For the imputation accuracy (r^2^), a 4-fold cross validation was used. Specifically, 4 groups of 50 individuals were selected randomly from the most recent generation to be used as the test groups. For each test group, the medium density genotypes were masked and imputation was used on the low-density genotypes using the remaining medium density training set. The accuracy of imputation was calculated as the correlation between the imputed genotypes and true genotypes for each SNP. The mean SNP accuracy was also calculated for each test group.

### Data and quality control

Ducks were reared according to standard commercial conditions using a starter feed for the first 14 days followed by a grower feed up to slaughter age. For photoperiodic regime, ducks were given 23 h of light on the first day, decreasing by an hour a day until 18 h light was reached. The birds then remained on 18 h of light until slaughter. Ducks were placed as hatched, so the sex ratio was 1:1. Ducks from each hatch within a generation were placed in the same house at two weekly intervals. Descriptive statistics are provided in Supplementary Table S5. Commercial ducks with both genotypic (MD) and phenotypic records were provided by Cherry Valley Farm (CVF) consisting of 1,445 ducks for one line. The available traits were: average daily gain (ADG), body weight (BW), primary feather length (PRF) and breast depth (BD). ADG was measured for variable periods between 2 and 6 weeks, we selected our ADG data during the period of 28–42 days. BW, PRF and BD were collected at 42 days of age. We explored the effect of body size on BD by fitting BW as a covariate in a linear mixed model thus creating a new trait of BDCOV. Especially for BD, it was measured using an ultrasound machine by placing the probe parallel to the keel bone at the deepest part of the breast muscle. The measurement was taken from the keel bone to the top of the muscle.

Animals were genotyped using a 60 K Affymetrix SNP array resulting in a total number of 59,870 autosomal SNPs. Quality control (QC) was performed in PLINK^43^ using call rate: 0.95 at a sample and marker level, MAF: 0.05 and HWE Fisher exact test with a cut-of p-value of 1.17492E-06. After QC, *n* = 1445 samples and 42,556 SNPs remained for further analyses.

The imputed data consisted of 13,020 ducks which had phenotypic records on the following same traits as already described above: ADG, BW, PRF and BD. The same QC filters were performed as for the medium density data were applied. After QC in PLINK^43^the following data was retained for further analyses: *n* = 13,020 samples and 46,297 autosomal SNPs.

### Univariate association analyses

Data genotyped with both medium density 60k SNP array and imputed to 60k genotypes were analyzed initially, pre-correcting for the fixed effects of sex and hatch in ASReml software using the following model:

1 $${\text{y}}_{{{\text{ij}}}} = \mu + {\text{H}}_{i} + {\text{S}}_{j} + {\text{e}}_{{ij}}$$

where y is the phenotype (ADG, BW, PRF, BD and BDCOV); µ is the overall mean; H_i_ is the effect of i^th^ hatch group (hatch 1–6); S_j_ the effect of j^th^ Sex (Male vs. Female) and e_ij_ being the residual error.

The pre-corrected phenotypes obtained from the above model (Model 1) were then used in subsequent variance component analysis in were then used in subsequent variance component analysis in ASReml software (Model 2):

2 $$y = \mu + {\mathbf{Z}}u + e$$

where y is the vector of pre-corrected phenotypes (ADG, BW, PRF, BD and BDCOV); µ is the overall mean; u and e are the vectors of additive polygenic and residuals effects, respectively, with incidence matrice **Z** for additive effects. The terms u and e were assumed to be normally distributed: N(0,Gσ^2^_a_) and N(0,Iσ^2^_e_) where G was the genomic relationship matrix computed from SNPs and I is the identity matrix and e is the error term.

The additive heritability estimates were calculated as additive variance divided by phenotypic variance (additive and residual variances) and GWAS using GEMMA^44^ software (Model 3) given below:

3 $$y = \mu + B_{k} M_{k} + {\mathbf{Z}}u + e$$

Same model as above but accounting for *β*_*i*_ as the regression coefficient of k^th^ marker, *m*_*k*_(1-42556 SNPs).

The same linear mixed models (2 and 3) were applied to both medium density and imputed data using GEMMA^44^. The Wald test statistic was used to infer the significant SNPs. In all GWAS analyses, the Bonferroni correction method was implemented to correct for multiple. In the MD data, GWAS results were considered to have achieved suggestive (1/number of SNPs used in the analysis) or genome-wide (0.05/number of SNPs used) significance level when SNPs had p-value < 2.349845e-05 or *p* < 1.174922e-06, respectively. On the other hand, in imputed data, the *p*-value < 2.159967e-05 and *p* < 1.079984e-06 were respectively suggestive and genome-wide significance threshold.

### Estimation of the genomic inflation factor

For every association analysis, the estimation of the genomic inflation factor (λ) was used to assess potential systematic bias due to population structure or the analytical approach^45^. If the λ value was greater than 1, it provided evidence for some systematic bias^45^. If the λ value was less than or equal to 1, no adjustment was needed^46^. Parameter λ was estimated using the estlambda function in GenABEL R package.

### Linkage disequilibrium (LD)

To investigate the underlying LD in the identified QTL regions within and across traits, we used the SNP pairwise correlation that was calculated using r^2^ in Plink 1.9 ^43^ and visualized in Haploview software^47^.

### Positional and functional candidate genes

Positional candidate genes were searched around those markers that were common between univariate GWA analyses with medium density and imputed data. We searched within 100 kb downstream and upstream flanking regions of each significant marker for positional candidate genes using the NCBI database and the ZJU1.0 assembly (https://www.ncbi.nlm.nih.gov/assembly/GCF_015476345.1, accessed 29 November 2024). Note here that physical positions of SNPs were initially based on a private genome assembly and thus we used the NCBI BLAST (http://www.ncbi.nlm.nih.gov/blast) nucleotide alignment against the ZJU1.0 assembly with 30 bases surrounding each marker. BLAST finds regions of local similarity between nucleotide sequences and calculates the statistical significance of the matches.

To specify the functional candidate genes, Gene Ontology (GO) Biological Process (BP) enrichment analysis was carried out using the ClueGO V2.5.7 ^48^ plug-in in Cytoscape V3.7.2 (http://cytoscape.org/^49^), . During this analysis, the GO BP annotations for *Anas platyrhynchos* were selected. Each time, the input genes were compared with a reference list of duck genes using the following settings: minimum number of genes per BP = 1, minimum percentage of genes included in GO BP = 3%, minimum GO levels = 3, maximum GO levels = 8 and minimum kappa score for GO BP network connectivity = 0.4. Here, GO BPs with FDR p-values lower than 0.05 for the right-sided hypergeometric test were considered as significantly enriched.

## Electronic supplementary material

Below is the link to the electronic supplementary material.

Supplementary Material 1

Supplementary Material 2

Supplementary Material 3

Supplementary Material 4

Supplementary Material 5

Supplementary Material 6

Supplementary Material 7

Supplementary Material 8

Supplementary Material 9

Supplementary Material 10

Supplementary Material 11

## Data Availability

The datasets generated during and/or analysed during the current study are not publicly available due to license agreement with Cherry Valley Farms (UK) Ltd, but are available from the corresponding author on reasonable request.
