# Supplementary material for: Leveraging genome-wide association analyses with chip and imputed data emerges potential pleiotropic region for four duck growth traits
Source: Sci Rep. 2025 Jul 2;15:23625. doi: 10.1038/s41598-025-08852-z (PMC12223076; doi:10.1038/s41598-025-08852-z)
Supplement: Supplementary file 1 — Supplementary Material 1 [file 41598_2025_8852_MOESM1_ESM.pdf]

Supplementary Table S1. Variance component analyses results from ASREML and heritability (h^2) with standard errors attached to estimates from GEMMA software using imputed data

Description	Traits				
	**BW**	**ADG**	**BD**	**BDCOV**	**PRF**
**ASREML Software**					
Number of records	13007	13007	11229	11241	11264
σ^2_a	15273±890	29.79±2.15	0.70±0.07	0.79±0.07	20.31±1.65
σ^2_e	26010±356	104.11±1.39	3.93± 0.06	3.52±0.05	84.41±1.21
σ^2_p	41284±882	133.91±2.31	4.63±0.08	4.31±0.07	104.72±1.82
h^2	0.37±0.01	0.22±0.01	0.15±0.01	0.18±0.01	0.19±0.01
**GEMMA Software**					
h^2	0.38±0.02	0.23±0.01	0.16±0.01	0.18±0.01	0.20±0.01

σ^2_a=additive genetic variance, σ^2_e= residual variance, σ^2_p= Phenotypic variance
