# Supplementary material for: Leveraging genome-wide association analyses with chip and imputed data emerges potential pleiotropic region for four duck growth traits
Source: Sci Rep. 2025 Jul 2;15:23625. doi: 10.1038/s41598-025-08852-z (PMC12223076; doi:10.1038/s41598-025-08852-z)
Supplement: Supplementary file 2 — Supplementary Material 2 [file 41598_2025_8852_MOESM2_ESM.pdf]

Supplementary Table S2. Genome-wide significant and suggestive SNPs for ADG, BW, PRF, BD and BDCOV using medium density data. Genome-wide significant SNPs are shown in bold.

Marker	Chr	Position (bp) 1	Start of marker's alignment (bp) 2	End of marker's alignment (bp) 2	P-value	Trait
**AX-399381139**	4	57590834	57743075	57743145	4.33E-15	ADG
**AX-399380966**	4	57576167	57727323	57727393	8.15E-15	ADG
**AX-391989849**	4	57583530	57735769	57735839	1.02E-14	ADG
**AX-406389417**	4	57496977	57648845	57648915	1.15E-14	ADG
**AX-399415535**	4	58780463	58922661	58922731	1.80E-14	ADG
**AX-399408144**	4	57890046	58041452	58041522	2.01E-14	ADG
**AX-399384273**	4	57770401	57921837	57921907	2.22E-14	ADG
**AX-391395264**	4	57874202	58025604	58025673	2.31E-14	ADG
**AX-399407752**	4	57869153	58020559	58020629	2.60E-14	ADG
**AX-399393735**	4	58713747	58855924	58855994	2.75E-14	ADG
**AX-399385210**	4	57818524	57969925	57969993	2.91E-14	ADG
**AX-399394817**	4	58884082	59026179	59026249	3.03E-14	ADG
**AX-399386541**	4	57883079	No significant similarity found by BLAST	No significant similarity found by BLAST	3.05E-14	ADG
**AX-406389915**	4	58840414	58982550	58982620	4.70E-14	ADG
**AX-247869802**	4	57859539	58010948	58011018	4.82E-14	ADG
**AX-413288581**	4	57617544	57769805	57769875	1.11E-13	ADG
**AX-399404909**	4	57744173	57895658	57895727	1.78E-13	ADG
**AX-399406303**	4	60406158	60609856	60609926	1.82E-12	ADG
**AX-399427267**	4	60395776	60599459	60599529	2.03E-12	ADG
**AX-399428424**	4	60516652	60720182	60720252	2.88E-12	ADG
**AX-399408850**	4	60599450	60802878	60802948	2.93E-12	ADG
**AX-399406221**	4	60389795	60593478	60593548	3.83E-12	ADG
**AX-399427636**	4	60439904	60643531	60643600	4.02E-12	ADG
**AX-399406282**	4	60401337	60605036	60605106	4.21E-12	ADG
**AX-399427492**	4	60423459	60627102	60627172	6.09E-12	ADG
**AX-399411060**	4	60711653	60915673	60915743	1.13E-11	ADG
**AX-399411115**	4	60720619	60924638	60924708	1.34E-11	ADG
**AX-399432307**	4	60738364	60942366	60942436	1.44E-11	ADG
**AX-399416702**	4	60729108	No significant similarity found by BLAST	No significant similarity found by BLAST	3.50E-11	ADG
**AX-391995514**	4	59520360	59679121	59679191	1.46E-10	ADG
**AX-247856762**	4	58809591	58951796	58951866	2.88E-10	ADG
**AX-396881416**	28	1257196	507906	507836	3.36E-10	ADG
**AX-391397463**	4	59406999	59565894	59565963	4.15E-10	ADG
**AX-399417739**	4	59264682	59423996	59424066	4.25E-10	ADG
**AX-247871298**	4	59782113	59987248	59987318	4.61E-10	ADG
**AX-399420827**	4	59663391	59822418	59822488	4.81E-10	ADG
**AX-391993165**	4	58753852	58896057	58896127	5.40E-10	ADG
**AX-399393490**	4	58693993	58836193	58836263	5.62E-10	ADG
**AX-391396574**	4	58838915	58981046	58981116	6.12E-10	ADG
**AX-399419051**	4	59410596	59569489	59569559	6.37E-10	ADG
**AX-399415377**	4	58758300	58900505	58900575	8.95E-10	ADG

**AX-399400325**	4	59742341	59947561	59947631	1.06E-09	ADG
**AX-399421434**	4	59747809	59953029	59953099	1.08E-09	ADG
**AX-247857157**	4	59375105	59534539	59534609	1.12E-09	ADG
**AX-399392532**	4	58527810	58669666	58669736	1.20E-09	ADG
**AX-391396430**	4	58736437	58878645	58878715	1.67E-09	ADG
**AX-396882625**	28	1286696	478564	478494	2.66E-09	ADG
**AX-399396415**	4	59227499	59386800	59386870	2.97E-09	ADG
**AX-399406967**	4	60475137	60678682	60678752	3.44E-09	ADG
**AX-391998834**	4	60555232	60758697	60758766	4.26E-09	ADG
**AX-396884834**	28	1339143	426112	426042	4.44E-09	ADG
**AX-396946052**	28	1269345	495752	495682	6.78E-09	ADG
**AX-247858317**	4	60642080	60845604	60845674	8.05E-09	ADG
**AX-396949886**	28	1362068	403202	403132	1.19E-08	ADG
**AX-396886496**	28	1383423	381847	381777	3.69E-08	ADG
**AX-399432939**	4	60803833	61007847	61007917	1.79E-07	ADG
**AX-399413292**	4	60866402	61070334	61070404	2.79E-07	ADG
**AX-399414599**	4	60943298	61147146	61147216	2.99E-07	ADG
**AX-399412691**	4	60839719	61043666	61043736	3.31E-07	ADG
**AX-399437119**	4	61085303	61289055	61289117	5.39E-07	ADG
**AX-247872616**	4	61090701	61294438	61294508	5.39E-07	ADG
**AX-399436319**	4	61001049	61204792	61204862	5.93E-07	ADG
**AX-399382231**	4	57671217	57823432	57823502	8.22E-07	ADG
**AX-399436070**	4	60972754	61176572	61176642	8.29E-07	ADG
**AX-247858741**	4	61032304	61236036	61236106	8.65E-07	ADG
**AX-399380444**	4	57540987	57692215	57692285	8.89E-07	ADG
**AX-399426907**	4	60352376	60556065	60556131	9.82E-07	ADG
**AX-391989691**	4	57536809	57688046	57688116	1.04E-06	ADG
**AX-391398482**	4	60078280	60283368	60283438	1.16E-06	ADG
AX-399425026	4	60159733	60364037	60364107	1.45E-06	ADG
AX-223726129	4	59909490	60114605	60114675	1.87E-06	ADG
AX-391997678	4	60203993	60408350	60408420	2.11E-06	ADG
AX-399384194	4	57765369	57916820	57916890	2.16E-06	ADG
AX-247858456	4	60769402	60973420	60973490	2.42E-06	ADG
AX-399408324	4	60557657	60761121	60761191	2.50E-06	ADG
AX-399402525	4	59978594	60183679	60183749	2.55E-06	ADG
AX-399431634	4	60676939	60880465	60880535	2.60E-06	ADG
AX-399410498	4	60672128	60875658	60875728	2.96E-06	ADG
AX-399387776	4	57960490	58111900	58111971	3.13E-06	ADG
AX-396872558	24	7528982	No significant similarity found by BLAST	No significant similarity found by BLAST	3.27E-06	ADG
AX-399429889	4	60601503	60804929	60804999	3.63E-06	ADG
AX-399416290	4	58959213	59101273	59101343	3.81E-06	ADG
AX-391396986	4	59138586	59280884	59280954	4.85E-06	ADG
AX-399388238	4	58032876	58184172	58184242	5.16E-06	ADG
AX-399390429	4	58338999	58480620	58480690	5.26E-06	ADG
AX-247871715	4	60239809	60443685	60443755	5.51E-06	ADG
AX-399409007	4	57947777	58099178	58099248	6.48E-06	ADG

AX-399387522	4	57941995	No significant similarity found by BLAST	No significant similarity found by BLAST	6.58E-06	ADG
AX-247871752	4	60275555	60479438	60479508	6.74E-06	ADG
AX-399432812	4	60794293	60998310	60998381	6.85E-06	ADG
AX-399403252	4	57648391	57800629	57800699	7.27E-06	ADG
AX-399399329	4	59597950	No significant similarity found by BLAST	No significant similarity found by BLAST	7.40E-06	ADG
AX-399412767	4	58419840	58561581	58561651	9.32E-06	ADG
AX-399416498	4	61166881	61370596	61370666	1.32E-05	ADG
**AX-409450699**	1	89659545	89355230	89355300	4.94E-10	BD
**AX-409596467**	12	3556621	No significant similarity found by BLAST	No significant similarity found by BLAST	9.08E-08	BD
AX-403641637	11	3533776	3842865	3842935	7.09E-06	BD
AX-412976359	3	39758727	No significant similarity found by BLAST	No significant similarity found by BLAST	7.84E-06	BD
AX-403004014	13	18541437	No significant similarity found by BLAST	No significant similarity found by BLAST	8.99E-06	BD
AX-404950114	19	9833857	No significant similarity found by BLAST	No significant similarity found by BLAST	1.25E-05	BD
AX-408272562	1	10861942	10571888	10571959	1.86E-05	BD
**AX-409450699**	1	89659545	89355230	89355300	1.01E-06	BDCOV
AX-409596467	12	3556621	No significant similarity found by BLAST	No significant similarity found by BLAST	3.10E-06	BDCOV
AX-411972909	21	4026804	No significant similarity found by BLAST	No significant similarity found by BLAST	1.59E-05	BDCOV
AX-409581163	12	20958162	883133	883063	2.06E-05	BDCOV
**AX-399384273**	4	57770401	57921837	57921907	3.33E-14	BW
**AX-399408144**	4	57890046	58041452	58041522	4.53E-14	BW
**AX-391395264**	4	57874202	58025604	58025673	5.65E-14	BW
**AX-399386541**	4	57883079	No significant similarity found by BLAST	No significant similarity found by BLAST	5.81E-14	BW
**AX-399407752**	4	57869153	58020559	58020629	5.82E-14	BW
**AX-399385210**	4	57818524	57969925	57969993	6.70E-14	BW
**AX-399393735**	4	58713747	58855924	58855994	6.82E-14	BW
**AX-399415535**	4	58780463	58922661	58922731	8.51E-14	BW
**AX-247869802**	4	57859539	58010948	58011018	1.31E-13	BW
**AX-399394817**	4	58884082	59026179	59026249	1.35E-13	BW
**AX-406389915**	4	58840414	58982550	58982620	2.66E-13	BW
**AX-391989849**	4	57583530	57735769	57735839	3.56E-13	BW

**AX-399381139**	4	57590834	57743075	57743145	9.25E-13	BW
**AX-399380966**	4	57576167	57727323	57727393	1.11E-12	BW
**AX-406389417**	4	57496977	57648845	57648915	4.10E-12	BW
**AX-399404909**	4	57744173	57895658	57895727	2.01E-11	BW
**AX-413288581**	4	57617544	57769805	57769875	6.57E-11	BW
**AX-399393490**	4	58693993	58836193	58836263	1.21E-10	BW
**AX-399415377**	4	58758300	58900505	58900575	1.92E-10	BW
**AX-391993165**	4	58753852	58896057	58896127	3.34E-10	BW
**AX-399420827**	4	59663391	59822418	59822488	3.54E-10	BW
**AX-391995514**	4	59520360	59679121	59679191	4.42E-10	BW
**AX-391396574**	4	58838915	58981046	58981116	5.85E-10	BW
**AX-399392532**	4	58527810	58669666	58669736	6.00E-10	BW
**AX-399417739**	4	59264682	59423996	59424066	6.05E-10	BW
**AX-247871298**	4	59782113	59987248	59987318	6.26E-10	BW
**AX-399427267**	4	60395776	60599459	60599529	6.63E-10	BW
**AX-399427492**	4	60423459	60627102	60627172	6.84E-10	BW
**AX-399427636**	4	60439904	60643531	60643600	7.16E-10	BW
**AX-247857157**	4	59375105	59534539	59534609	7.78E-10	BW
**AX-247856762**	4	58809591	58951796	58951866	8.46E-10	BW
**AX-399400325**	4	59742341	59947561	59947631	8.64E-10	BW
**AX-399419051**	4	59410596	59569489	59569559	9.08E-10	BW
**AX-391397463**	4	59406999	59565894	59565963	9.35E-10	BW
**AX-399406221**	4	60389795	60593478	60593548	1.07E-09	BW
**AX-399408850**	4	60599450	60802878	60802948	1.08E-09	BW
**AX-399428424**	4	60516652	60720182	60720252	1.15E-09	BW
**AX-399406303**	4	60406158	60609856	60609926	1.16E-09	BW
**AX-399411060**	4	60711653	60915673	60915743	1.30E-09	BW
**AX-399416702**	4	60729108	No significant similarity found by BLAST	No significant similarity found by BLAST	1.36E-09	BW
**AX-391396430**	4	58736437	58878645	58878715	1.54E-09	BW
**AX-399421434**	4	59747809	59953029	59953099	1.67E-09	BW
**AX-399406282**	4	60401337	60605036	60605106	1.80E-09	BW
**AX-399432307**	4	60738364	60942366	60942436	2.45E-09	BW
**AX-399411115**	4	60720619	60924638	60924708	2.47E-09	BW
**AX-399396415**	4	59227499	59386800	59386870	1.77E-08	BW
**AX-399387776**	4	57960490	58111900	58111971	6.25E-08	BW
**AX-396881416**	28	1257196	507906	507836	1.06E-07	BW
**AX-399390429**	4	58338999	58480620	58480690	1.09E-07	BW
**AX-399409007**	4	57947777	58099178	58099248	1.11E-07	BW
**AX-396946052**	28	1269345	495752	495682	1.18E-07	BW
**AX-399388238**	4	58032876	58184172	58184242	1.43E-07	BW
**AX-399387522**	4	57941995	No significant similarity found by BLAST	No significant similarity found by BLAST	1.52E-07	BW
**AX-399412767**	4	58419840	58561581	58561651	2.35E-07	BW
**AX-396884834**	28	1339143	426112	426042	2.92E-07	BW
**AX-396886496**	28	1383423	381847	381777	4.23E-07	BW
**AX-399406967**	4	60475137	60678682	60678752	4.60E-07	BW
**AX-396882625**	28	1286696	478564	478494	4.81E-07	BW

**AX-396872558**	24	7528982	No significant similarity found by BLAST	No significant similarity found by BLAST	4.90E-07	BW
**AX-247858317**	4	60642080	60845604	60845674	8.04E-07	BW
**AX-391998834**	4	60555232	60758697	60758766	8.23E-07	BW
**AX-399432812**	4	60794293	60998310	60998381	8.82E-07	BW
**AX-396949886**	28	1362068	403202	403132	1.04E-06	BW
**AX-399425026**	4	60159733	60364037	60364107	1.08E-06	BW
**AX-399416290**	4	58959213	59101273	59101343	1.11E-06	BW
AX-223726129	4	59909490	60114605	60114675	1.45E-06	BW
AX-399426907	4	60352376	60556065	60556131	2.54E-06	BW
AX-391396986	4	59138586	59280884	59280954	3.29E-06	BW
AX-391398482	4	60078280	60283368	60283438	3.47E-06	BW
AX-399402525	4	59978594	60183679	60183749	6.60E-06	BW
AX-399431634	4	60676939	60880465	60880535	8.58E-06	BW
AX-399408324	4	60557657	60761121	60761191	8.82E-06	BW
AX-399399329	4	59597950	No significant similarity found by BLAST	No significant similarity found by BLAST	9.27E-06	BW
AX-399410498	4	60672128	60875658	60875728	9.55E-06	BW
AX-247871752	4	60275555	60479438	60479508	9.79E-06	BW
AX-413288808	4	58149809	58291447	58291517	1.07E-05	BW
AX-247857972	4	60313959	60517874	60517944	1.17E-05	BW
AX-247858456	4	60769402	60973420	60973490	1.24E-05	BW
AX-399387025	4	57909625	58061038	58061109	1.25E-05	BW
AX-399409883	4	58063334	58214625	58214689	1.25E-05	BW
AX-399388585	4	58073103	58224363	58224433	1.25E-05	BW
AX-391992216	4	58458842	58600535	58600605	1.42E-05	BW
AX-247871715	4	60239809	60443685	60443755	1.57E-05	BW
AX-399429889	4	60601503	60804929	60804999	1.77E-05	BW
AX-223956510	4	52714660	52845614	52845684	1.94E-05	BW
AX-391396693	4	58926599	59068682	59068750	1.97E-05	BW
**AX-406389417**	4	57496977	57648845	57648915	1.05E-07	PRF
**AX-399380966**	4	57576167	57727323	57727393	1.12E-07	PRF
**AX-399394817**	4	58884082	59026179	59026249	1.13E-07	PRF
**AX-413288581**	4	57617544	57769805	57769875	1.33E-07	PRF
**AX-399381139**	4	57590834	57743075	57743145	1.37E-07	PRF
**AX-399415535**	4	58780463	58922661	58922731	1.42E-07	PRF
**AX-399404909**	4	57744173	57895658	57895727	1.43E-07	PRF
**AX-406389915**	4	58840414	58982550	58982620	1.59E-07	PRF
**AX-399393735**	4	58713747	58855924	58855994	2.12E-07	PRF
**AX-247869802**	4	57859539	58010948	58011018	2.20E-07	PRF
**AX-391395264**	4	57874202	58025604	58025673	2.22E-07	PRF
**AX-399385210**	4	57818524	57969925	57969993	2.29E-07	PRF
**AX-399407752**	4	57869153	58020559	58020629	2.50E-07	PRF
**AX-399408144**	4	57890046	58041452	58041522	2.51E-07	PRF
**AX-399386541**	4	57883079	No significant similarity found by BLAST	No significant similarity found by BLAST	3.53E-07	PRF
**AX-391995514**	4	59520360	59679121	59679191	4.95E-07	PRF

**AX-391397463**	4	59406999	59565894	59565963	5.66E-07	PRF
**AX-399396415**	4	59227499	59386800	59386870	6.51E-07	PRF
**AX-247857157**	4	59375105	59534539	59534609	6.67E-07	PRF
**AX-247871298**	4	59782113	59987248	59987318	7.66E-07	PRF
**AX-399420827**	4	59663391	59822418	59822488	9.88E-07	PRF
**AX-399421434**	4	59747809	59953029	59953099	9.90E-07	PRF
**AX-399417739**	4	59264682	59423996	59424066	9.98E-07	PRF
**AX-391989849**	4	57583530	57735769	57735839	1.15E-06	PRF
AX-399400325	4	59742341	59947561	59947631	1.23E-06	PRF
AX-399419051	4	59410596	59569489	59569559	1.23E-06	PRF
AX-399393490	4	58693993	58836193	58836263	1.36E-06	PRF
AX-399384273	4	57770401	57921837	57921907	1.49E-06	PRF
AX-391993165	4	58753852	58896057	58896127	1.65E-06	PRF
AX-391396574	4	58838915	58981046	58981116	1.66E-06	PRF
AX-247856762	4	58809591	58951796	58951866	2.04E-06	PRF
AX-399392532	4	58527810	58669666	58669736	2.14E-06	PRF
AX-391997678	4	60203993	60408350	60408420	2.67E-06	PRF
AX-391396430	4	58736437	58878645	58878715	3.42E-06	PRF
AX-399382231	4	57671217	57823432	57823502	4.41E-06	PRF
AX-399415377	4	58758300	58900505	58900575	4.47E-06	PRF
AX-405356168	18	9772289	9805323	9805393	4.54E-06	PRF
AX-393654576	18	9463473	9496539	9496609	4.71E-06	PRF
AX-399428424	4	60516652	60720182	60720252	6.04E-06	PRF
AX-399427267	4	60395776	60599459	60599529	7.37E-06	PRF
AX-399406303	4	60406158	60609856	60609926	8.03E-06	PRF
AX-412249871	18	9777793	9810810	9810880	8.85E-06	PRF
AX-399408850	4	60599450	60802878	60802948	9.12E-06	PRF
AX-399406282	4	60401337	60605036	60605106	9.28E-06	PRF
AX-399427492	4	60423459	60627102	60627172	9.65E-06	PRF
AX-399406221	4	60389795	60593478	60593548	1.20E-05	PRF
AX-399411060	4	60711653	60915673	60915743	1.28E-05	PRF
AX-399427636	4	60439904	60643531	60643600	1.37E-05	PRF
AX-391995141	4	59391254	59550168	59550238	1.45E-05	PRF
AX-391995289	4	59437092	59595963	59596033	1.67E-05	PRF
AX-399411115	4	60720619	60924638	60924708	1.70E-05	PRF
AX-399432307	4	60738364	60942366	60942436	1.73E-05	PRF
AX-405357425	18	9843468	9876585	9876655	1.77E-05	PRF
AX-399380444	4	57540987	57692215	57692285	1.77E-05	PRF
AX-393656608	18	9842030	9875146	9875216	1.85E-05	PRF
AX-391397334	4	59342799	59502015	59502082	1.96E-05	PRF
AX-248256594	28	240136	No significant similarity found by BLAST	No significant similarity found by BLAST	2.09E-05	PRF

1 Positions of SNPs that were initially based on a private genome assembly.

2 Positions of SNPs that were based on ZJU1.0 assembly using NCBI BLAST.
