# Supplementary material for: Leveraging genome-wide association analyses with chip and imputed data emerges potential pleiotropic region for four duck growth traits
Source: Sci Rep. 2025 Jul 2;15:23625. doi: 10.1038/s41598-025-08852-z (PMC12223076; doi:10.1038/s41598-025-08852-z)
Supplement: Supplementary file 3 — Supplementary Material 3 [file 41598_2025_8852_MOESM3_ESM.pdf]

Supplementary Table S3. Genome-wide significant and suggestive SNPs for ADG, BW, PRF, BD and BDCOV using imputed data.
Genome-wide significant SNPs are shown in bold.

Marker	Chr	Position (bp) 1	Start of marker's alignment (bp) 2	End of marker's alignment (bp) 2	P-value	Trait
**AX-406389915**	4	58840414	58982550	58982620	3.62E-33	ADG
**AX-399394817**	4	58884082	59026179	59026249	3.62E-33	ADG
**AX-399415535**	4	58780463	58922661	58922731	7.32E-32	ADG
**AX-399393735**	4	58713747	58855924	58855994	1.84E-31	ADG
**AX-399386541**	4	57883079	No significant similarity found by BLAST	No significant similarity found by BLAST	7.51E-31	ADG
**AX-399408144**	4	57890046	58041452	58041522	9.47E-31	ADG
**AX-391395264**	4	57874202	58025604	58025673	2.07E-30	ADG
**AX-247869802**	4	57859539	58010948	58011018	3.25E-30	ADG
**AX-399407752**	4	57869153	58020559	58020629	3.25E-30	ADG
**AX-406389417**	4	57496977	57648845	57648915	4.14E-30	ADG
**AX-399385210**	4	57818524	57969925	57969993	1.08E-29	ADG
**AX-413288581**	4	57617544	57769805	57769875	9.38E-29	ADG
**AX-399381139**	4	57590834	57743075	57743145	1.46E-28	ADG
**AX-399380966**	4	57576167	57727323	57727393	1.58E-28	ADG
**AX-399404909**	4	57744173	57895658	57895727	2.98E-28	ADG
**AX-391989849**	4	57583530	57735769	57735839	4.05E-27	ADG
**AX-399384273**	4	57770401	57921837	57921907	7.41E-27	ADG
**AX-399396415**	4	59227499	59386800	59386870	4.62E-23	ADG
**AX-399417739**	4	59264682	59423996	59424066	7.47E-23	ADG
**AX-399400325**	4	59742341	59947561	59947631	3.94E-22	ADG
**AX-399420827**	4	59663391	59822418	59822488	4.50E-22	ADG
**AX-399421434**	4	59747809	59953029	59953099	4.87E-22	ADG
**AX-247857157**	4	59375105	59534539	59534609	6.27E-22	ADG
**AX-247856762**	4	58809591	58951796	58951866	6.55E-22	ADG
**AX-391396574**	4	58838915	58981046	58981116	6.55E-22	ADG
**AX-247871298**	4	59782113	59987248	59987318	6.65E-22	ADG
**AX-391397463**	4	59406999	59565894	59565963	1.35E-21	ADG
**AX-399419051**	4	59410596	59569489	59569559	1.35E-21	ADG
**AX-391995514**	4	59520360	59679121	59679191	1.45E-21	ADG
**AX-391396430**	4	58736437	58878645	58878715	6.62E-21	ADG
**AX-391993165**	4	58753852	58896057	58896127	6.62E-21	ADG
**AX-399415377**	4	58758300	58900505	58900575	6.65E-21	ADG
**AX-399393490**	4	58693993	58836193	58836263	7.66E-21	ADG
**AX-399392532**	4	58527810	58669666	58669736	1.06E-20	ADG
**AX-399406221**	4	60389795	60593478	60593548	1.20E-19	ADG
**AX-399427267**	4	60395776	60599459	60599529	1.26E-19	ADG
**AX-399406282**	4	60401337	60605036	60605106	1.31E-19	ADG
**AX-399406303**	4	60406158	60609856	60609926	1.31E-19	ADG
**AX-399427492**	4	60423459	60627102	60627172	1.31E-19	ADG
**AX-399428424**	4	60516652	60720182	60720252	1.53E-19	ADG
**AX-399427636**	4	60439904	60643531	60643600	1.81E-19	ADG
**AX-399408850**	4	60599450	60802878	60802948	4.63E-19	ADG
**AX-391997678**	4	60203993	60408350	60408420	3.72E-17	ADG

**AX-399411060**	4	60711653	60915673	60915743	3.79E-17	ADG
**AX-399411115**	4	60720619	60924638	60924708	3.79E-17	ADG
**AX-399416702**	4	60729108	No significant similarity found by BLAST	No significant similarity found by BLAST	3.79E-17	ADG
**AX-399432307**	4	60738364	60942366	60942436	3.79E-17	ADG
**AX-399432939**	4	60803833	61007847	61007917	3.78E-15	ADG
**AX-399412691**	4	60839719	61043666	61043736	3.78E-15	ADG
**AX-399413292**	4	60866402	61070334	61070404	4.66E-15	ADG
**AX-391999771**	4	60874627	No significant similarity found by BLAST	No significant similarity found by BLAST	5.56E-15	ADG
**AX-391989691**	4	57536809	57688046	57688116	7.12E-15	ADG
**AX-399414599**	4	60943298	61147146	61147216	7.12E-15	ADG
**AX-399380444**	4	57540987	57692215	57692285	1.02E-14	ADG
**AX-399436319**	4	61001049	61204792	61204862	2.56E-14	ADG
**AX-247858741**	4	61032304	61236036	61236106	2.56E-14	ADG
**AX-399382231**	4	57671217	57823432	57823502	2.99E-14	ADG
**AX-399437119**	4	61085303	61289055	61289117	3.02E-14	ADG
**AX-247872616**	4	61090701	61294438	61294508	3.02E-14	ADG
**AX-399403252**	4	57648391	57800629	57800699	6.25E-14	ADG
**AX-399436070**	4	60972754	61176572	61176642	7.07E-14	ADG
**AX-399384194**	4	57765369	57916820	57916890	7.12E-14	ADG
**AX-391995141**	4	59391254	59550168	59550238	9.05E-12	ADG
**AX-391995289**	4	59437092	59595963	59596033	9.05E-12	ADG
**AX-391397334**	4	59342799	59502015	59502082	1.01E-11	ADG
**AX-399418571**	4	59350770	59510080	59510150	1.01E-11	ADG
**AX-399421778**	4	59799292	No significant similarity found by BLAST	No significant similarity found by BLAST	2.50E-11	ADG
**AX-391998834**	4	60555232	60758697	60758766	4.45E-11	ADG
**AX-399406967**	4	60475137	60678682	60678752	4.57E-11	ADG
**AX-406390235**	4	59676746	59838652	59838722	5.10E-11	ADG
**AX-247858317**	4	60642080	60845604	60845674	1.82E-10	ADG
**AX-391397176**	4	59245202	No significant similarity found by BLAST	No significant similarity found by BLAST	2.28E-10	ADG
**AX-399416498**	4	61166881	61370596	61370666	2.84E-10	ADG
**AX-396951253**	28	1420070	345241	345171	6.76E-09	ADG
**AX-247858859**	4	61134862	61338569	61338639	1.71E-08	ADG
**AX-399416216**	4	61119870	61323593	61323663	4.04E-08	ADG
**AX-391398482**	4	60078280	60283368	60283438	3.71E-07	ADG
**AX-399415693**	4	61063742	61267493	61267563	4.22E-07	ADG
**AX-399402525**	4	59978594	60183679	60183749	5.20E-07	ADG
**AX-399414097**	4	60910462	61114365	61114435	5.89E-07	ADG
**AX-399425026**	4	60159733	60364037	60364107	5.96E-07	ADG
**AX-223726129**	4	59909490	60114605	60114675	7.15E-07	ADG
**AX-399426907**	4	60352376	60556065	60556131	1.08E-06	ADG
AX-247871715	4	60239809	60443685	60443755	1.17E-06	ADG
AX-247871752	4	60275555	60479438	60479508	1.17E-06	ADG

AX-399317638	4	52489177	No significant similarity found by BLAST	No significant similarity found by BLAST	1.59E-06	ADG
AX-399429889	4	60601503	60804929	60804999	1.72E-06	ADG
AX-391396986	4	59138586	59280884	59280954	1.85E-06	ADG
AX-399408324	4	60557657	60761121	60761191	1.88E-06	ADG
AX-247858456	4	60769402	60973420	60973490	1.95E-06	ADG
AX-399410498	4	60672128	60875658	60875728	2.13E-06	ADG
AX-399431634	4	60676939	60880465	60880535	2.13E-06	ADG
AX-399432812	4	60794293	60998310	60998381	2.18E-06	ADG
AX-399399329	4	59597950	No significant similarity found by BLAST	No significant similarity found by BLAST	2.19E-06	ADG
AX-399416290	4	58959213	59101273	59101343	2.28E-06	ADG
AX-399409007	4	57947777	58099178	58099248	6.19E-06	ADG
AX-399323347	4	52823066	52954053	52954122	6.53E-06	ADG
AX-399387776	4	57960490	58111900	58111971	8.32E-06	ADG
AX-399387522	4	57941995	No significant similarity found by BLAST	No significant similarity found by BLAST	1.08E-05	ADG
AX-397193416	28	818519	No significant similarity found by BLAST	No significant similarity found by BLAST	1.50E-05	ADG
AX-247550894	1	204714710	No significant similarity found by BLAST	No significant similarity found by BLAST	1.52E-05	ADG
AX-399340029	4	52482614	52613457	52613527	1.68E-05	ADG
AX-391972893	4	52495712	52626516	52626586	1.68E-05	ADG
AX-399314752	4	52323210	52453969	52454039	2.00E-05	ADG
AX-399388238	4	58032876	58184172	58184242	2.05E-05	ADG
**AX-397878483**	20	3050584	No significant similarity found by BLAST	No significant similarity found by BLAST	2.62E-07	BD
AX-398500103	Z	42123288	No significant similarity found by BLAST	No significant similarity found by BLAST	8.22E-06	BD
AX-398506837	Z	43595172	No significant similarity found by BLAST	No significant similarity found by BLAST	8.82E-06	BD
AX-391098998	2	236497	No significant similarity found by BLAST	No significant similarity found by BLAST	1.45E-05	BD
AX-404056312	14	9588693	No significant similarity found by BLAST	No significant similarity found by BLAST	1.84E-05	BD
**AX-247871298**	4	59782113	59987248	59987318	3.38E-07	BDCOV
**AX-391397463**	4	59406999	59565894	59565963	3.68E-07	BDCOV

**AX-399419051**	4	59410596	59569489	59569559	3.68E-07	BDCOV
**AX-399421434**	4	59747809	59953029	59953099	3.91E-07	BDCOV
**AX-247857157**	4	59375105	59534539	59534609	4.56E-07	BDCOV
**AX-399400325**	4	59742341	59947561	59947631	4.68E-07	BDCOV
**AX-399420827**	4	59663391	59822418	59822488	6.28E-07	BDCOV
**AX-397878483**	20	3050584	No significant similarity found by BLAST	No significant similarity found by BLAST	6.66E-07	BDCOV
**AX-391995514**	4	59520360	59679121	59679191	7.12E-07	BDCOV
**AX-398506837**	Z	43595172	No significant similarity found by BLAST	No significant similarity found by BLAST	8.04E-07	BDCOV
**AX-406389417**	4	57496977	57648845	57648915	8.30E-07	BDCOV
**AX-398500103**	Z	42123288	No significant similarity found by BLAST	No significant similarity found by BLAST	9.14E-07	BDCOV
**AX-399411060**	4	60711653	60915673	60915743	9.33E-07	BDCOV
**AX-399411115**	4	60720619	60924638	60924708	9.33E-07	BDCOV
**AX-399416702**	4	60729108	No significant similarity found by BLAST	No significant similarity found by BLAST	9.33E-07	BDCOV
**AX-399432307**	4	60738364	60942366	60942436	9.33E-07	BDCOV
AX-399417739	4	59264682	59423996	59424066	1.21E-06	BDCOV
AX-399428424	4	60516652	60720182	60720252	1.25E-06	BDCOV
AX-399396415	4	59227499	59386800	59386870	1.26E-06	BDCOV
AX-399427267	4	60395776	60599459	60599529	1.38E-06	BDCOV
AX-247856762	4	58809591	58951796	58951866	1.45E-06	BDCOV
AX-391396574	4	58838915	58981046	58981116	1.45E-06	BDCOV
AX-399406221	4	60389795	60593478	60593548	1.60E-06	BDCOV
AX-399384273	4	57770401	57921837	57921907	1.70E-06	BDCOV
AX-399406282	4	60401337	60605036	60605106	1.75E-06	BDCOV

AX-399406303	4	60406158	60609856	60609926	1.75E-06	BDCOV
AX-399427492	4	60423459	60627102	60627172	1.75E-06	BDCOV
AX-406389915	4	58840414	58982550	58982620	1.75E-06	BDCOV
AX-399394817	4	58884082	59026179	59026249	1.75E-06	BDCOV
AX-399415377	4	58758300	58900505	58900575	1.80E-06	BDCOV
AX-399427636	4	60439904	60643531	60643600	2.08E-06	BDCOV
AX-399393490	4	58693993	58836193	58836263	2.22E-06	BDCOV
AX-391396430	4	58736437	58878645	58878715	2.36E-06	BDCOV
AX-391993165	4	58753852	58896057	58896127	2.36E-06	BDCOV
AX-399415535	4	58780463	58922661	58922731	2.36E-06	BDCOV
AX-391989849	4	57583530	57735769	57735839	2.54E-06	BDCOV
AX-399393735	4	58713747	58855924	58855994	2.95E-06	BDCOV
AX-399408850	4	60599450	60802878	60802948	3.05E-06	BDCOV
AX-399392532	4	58527810	58669666	58669736	3.08E-06	BDCOV
AX-399385210	4	57818524	57969925	57969993	5.85E-06	BDCOV
AX-406390235	4	59676746	59838652	59838722	9.76E-06	BDCOV
AX-399404909	4	57744173	57895658	57895727	9.77E-06	BDCOV
AX-247869802	4	57859539	58010948	58011018	1.03E-05	BDCOV
AX-399407752	4	57869153	58020559	58020629	1.03E-05	BDCOV
AX-399381139	4	57590834	57743075	57743145	1.26E-05	BDCOV
AX-399408144	4	57890046	58041452	58041522	1.31E-05	BDCOV
AX-391395264	4	57874202	58025604	58025673	1.39E-05	BDCOV
AX-413288581	4	57617544	57769805	57769875	1.40E-05	BDCOV
AX-399386541	4	57883079	No significant similarity found by BLAST	No significant similarity found by BLAST	1.48E-05	BDCOV

AX-399380966	4	57576167	57727323	57727393	1.54E-05	BDCOV
AX-399421778	4	59799292	No significant similarity found by BLAST	No significant similarity found by BLAST	1.69E-05	BDCOV
AX-398407145	Z	28748651	No significant similarity found by BLAST	No significant similarity found by BLAST	1.74E-05	BDCOV
AX-248161300	16	13873633	No significant similarity found by BLAST	No significant similarity found by BLAST	1.92E-05	BDCOV
**AX-406389417**	4	57496977	57648845	57648915	5.79E-19	BW
**AX-399386541**	4	57883079	No significant similarity found by BLAST	No significant similarity found by BLAST	1.10E-17	BW
**AX-399408144**	4	57890046	58041452	58041522	1.13E-17	BW
**AX-391395264**	4	57874202	58025604	58025673	1.47E-17	BW
**AX-247869802**	4	57859539	58010948	58011018	2.05E-17	BW
**AX-399407752**	4	57869153	58020559	58020629	2.05E-17	BW
**AX-399415535**	4	58780463	58922661	58922731	2.16E-17	BW
**AX-399385210**	4	57818524	57969925	57969993	5.77E-17	BW
**AX-399393735**	4	58713747	58855924	58855994	8.42E-17	BW
**AX-406389915**	4	58840414	58982550	58982620	2.16E-16	BW
**AX-399394817**	4	58884082	59026179	59026249	2.16E-16	BW
**AX-399404909**	4	57744173	57895658	57895727	3.20E-16	BW
**AX-399380966**	4	57576167	57727323	57727393	4.24E-16	BW
**AX-399381139**	4	57590834	57743075	57743145	9.43E-16	BW
**AX-413288581**	4	57617544	57769805	57769875	9.46E-16	BW
**AX-391989849**	4	57583530	57735769	57735839	2.02E-15	BW
**AX-399384273**	4	57770401	57921837	57921907	2.75E-15	BW
**AX-399393490**	4	58693993	58836193	58836263	7.50E-12	BW
**AX-399396415**	4	59227499	59386800	59386870	9.76E-12	BW
**AX-399415377**	4	58758300	58900505	58900575	1.15E-11	BW
**AX-391396430**	4	58736437	58878645	58878715	1.19E-11	BW
**AX-391993165**	4	58753852	58896057	58896127	1.19E-11	BW
**AX-399417739**	4	59264682	59423996	59424066	1.21E-11	BW
**AX-399392532**	4	58527810	58669666	58669736	1.33E-11	BW
**AX-247871298**	4	59782113	59987248	59987318	1.65E-11	BW
**AX-399420827**	4	59663391	59822418	59822488	1.94E-11	BW
**AX-399400325**	4	59742341	59947561	59947631	2.00E-11	BW
**AX-399421434**	4	59747809	59953029	59953099	2.20E-11	BW
**AX-247857157**	4	59375105	59534539	59534609	2.73E-11	BW
**AX-391397463**	4	59406999	59565894	59565963	3.36E-11	BW
**AX-399419051**	4	59410596	59569489	59569559	3.36E-11	BW
**AX-247856762**	4	58809591	58951796	58951866	5.89E-11	BW
**AX-391396574**	4	58838915	58981046	58981116	5.89E-11	BW
**AX-391995514**	4	59520360	59679121	59679191	6.15E-11	BW
**AX-396951253**	28	1420070	345241	345171	1.25E-09	BW
**AX-391997678**	4	60203993	60408350	60408420	5.37E-09	BW
**AX-399408850**	4	60599450	60802878	60802948	1.15E-07	BW

**AX-399427267**	4	60395776	60599459	60599529	2.05E-07	BW
**AX-399406221**	4	60389795	60593478	60593548	2.06E-07	BW
**AX-399427636**	4	60439904	60643531	60643600	2.37E-07	BW
**AX-399406282**	4	60401337	60605036	60605106	2.70E-07	BW
**AX-399406303**	4	60406158	60609856	60609926	2.70E-07	BW
**AX-399427492**	4	60423459	60627102	60627172	2.70E-07	BW
**AX-399432939**	4	60803833	61007847	61007917	4.92E-07	BW
**AX-399412691**	4	60839719	61043666	61043736	4.92E-07	BW
**AX-399428424**	4	60516652	60720182	60720252	6.77E-07	BW
**AX-399414599**	4	60943298	61147146	61147216	7.64E-07	BW
**AX-399413292**	4	60866402	61070334	61070404	8.59E-07	BW
**AX-391999771**	4	60874627	No significant similarity found by BLAST	No significant similarity found by BLAST	1.01E-06	BW
**AX-399437119**	4	61085303	61289055	61289117	1.05E-06	BW
**AX-247872616**	4	61090701	61294438	61294508	1.05E-06	BW
AX-399436319	4	61001049	61204792	61204862	1.26E-06	BW
AX-247858741	4	61032304	61236036	61236106	1.26E-06	BW
AX-399421778	4	59799292	No significant similarity found by BLAST	No significant similarity found by BLAST	1.38E-06	BW
AX-399411060	4	60711653	60915673	60915743	2.18E-06	BW
AX-399411115	4	60720619	60924638	60924708	2.18E-06	BW
AX-399416702	4	60729108	No significant similarity found by BLAST	No significant similarity found by BLAST	2.18E-06	BW
AX-399432307	4	60738364	60942366	60942436	2.18E-06	BW
AX-391995141	4	59391254	59550168	59550238	2.98E-06	BW
AX-391995289	4	59437092	59595963	59596033	2.98E-06	BW
AX-391989691	4	57536809	57688046	57688116	3.22E-06	BW
AX-247431082	1	53653547	No significant similarity found by BLAST	No significant similarity found by BLAST	3.37E-06	BW
AX-406390235	4	59676746	59838652	59838722	3.46E-06	BW
AX-399436070	4	60972754	61176572	61176642	3.53E-06	BW
AX-391397334	4	59342799	59502015	59502082	3.89E-06	BW
AX-399418571	4	59350770	59510080	59510150	3.89E-06	BW
AX-391972227	4	52186796	52317577	52317647	4.30E-06	BW
AX-399312800	4	52193485	52324246	52324316	1.03E-05	BW
AX-399380444	4	57540987	57692215	57692285	1.19E-05	BW
AX-247864522	4	52195082	52325843	52325913	1.61E-05	BW
AX-247858859	4	61134862	61338569	61338639	2.08E-05	BW
**AX-406389915**	4	58840414	58982550	58982620	1.55E-10	PRF
**AX-399394817**	4	58884082	59026179	59026249	1.55E-10	PRF
**AX-399415535**	4	58780463	58922661	58922731	2.65E-10	PRF
**AX-399393735**	4	58713747	58855924	58855994	3.83E-10	PRF
**AX-399386541**	4	57883079	No significant similarity found by BLAST	No significant similarity found by BLAST	4.54E-09	PRF
**AX-391395264**	4	57874202	58025604	58025673	5.62E-09	PRF

**AX-399408144**	4	57890046	58041452	58041522	5.70E-09	PRF
**AX-406389417**	4	57496977	57648845	57648915	8.34E-09	PRF
**AX-247869802**	4	57859539	58010948	58011018	1.05E-08	PRF
**AX-399407752**	4	57869153	58020559	58020629	1.05E-08	PRF
**AX-399385210**	4	57818524	57969925	57969993	1.49E-08	PRF
**AX-413288581**	4	57617544	57769805	57769875	2.94E-08	PRF
**AX-399404909**	4	57744173	57895658	57895727	3.13E-08	PRF
**AX-399380966**	4	57576167	57727323	57727393	3.44E-08	PRF
**AX-391995514**	4	59520360	59679121	59679191	3.45E-08	PRF
**AX-247856762**	4	58809591	58951796	58951866	3.88E-08	PRF
**AX-391396574**	4	58838915	58981046	58981116	3.88E-08	PRF
**AX-399396415**	4	59227499	59386800	59386870	4.45E-08	PRF
**AX-402935795**	13	15360267	No significant similarity found by BLAST	No significant similarity found by BLAST	4.52E-08	PRF
**AX-399381139**	4	57590834	57743075	57743145	4.87E-08	PRF
**AX-399417739**	4	59264682	59423996	59424066	5.07E-08	PRF
**AX-391999771**	4	60874627	No significant similarity found by BLAST	No significant similarity found by BLAST	7.01E-08	PRF
**AX-399393490**	4	58693993	58836193	58836263	7.21E-08	PRF
**AX-399415377**	4	58758300	58900505	58900575	7.22E-08	PRF
**AX-391396430**	4	58736437	58878645	58878715	9.25E-08	PRF
**AX-391993165**	4	58753852	58896057	58896127	9.25E-08	PRF
**AX-247857157**	4	59375105	59534539	59534609	1.07E-07	PRF
**AX-399392532**	4	58527810	58669666	58669736	1.10E-07	PRF
**AX-399384273**	4	57770401	57921837	57921907	1.51E-07	PRF
**AX-391397463**	4	59406999	59565894	59565963	1.52E-07	PRF
**AX-399419051**	4	59410596	59569489	59569559	1.52E-07	PRF
**AX-391989849**	4	57583530	57735769	57735839	1.78E-07	PRF
**AX-399420827**	4	59663391	59822418	59822488	2.59E-07	PRF
**AX-399400325**	4	59742341	59947561	59947631	3.01E-07	PRF
**AX-397193416**	28	818519	No significant similarity found by BLAST	No significant similarity found by BLAST	3.29E-07	PRF
**AX-391997678**	4	60203993	60408350	60408420	4.00E-07	PRF
**AX-399432939**	4	60803833	61007847	61007917	5.30E-07	PRF
**AX-399412691**	4	60839719	61043666	61043736	5.30E-07	PRF
**AX-399413292**	4	60866402	61070334	61070404	5.57E-07	PRF
**AX-399421434**	4	59747809	59953029	59953099	5.92E-07	PRF
**AX-399414599**	4	60943298	61147146	61147216	7.58E-07	PRF
**AX-247871298**	4	59782113	59987248	59987318	8.44E-07	PRF
AX-399436319	4	61001049	61204792	61204862	1.60E-06	PRF
AX-247858741	4	61032304	61236036	61236106	1.60E-06	PRF
AX-399437119	4	61085303	61289055	61289117	1.69E-06	PRF
AX-247872616	4	61090701	61294438	61294508	1.69E-06	PRF
AX-399436070	4	60972754	61176572	61176642	2.40E-06	PRF
AX-391397176	4	59245202	No significant similarity found by BLAST	No significant similarity found by BLAST	4.18E-06	PRF

AX-399406282	4	60401337	60605036	60605106	7.18E-06	PRF
AX-399406303	4	60406158	60609856	60609926	7.18E-06	PRF
AX-399427492	4	60423459	60627102	60627172	7.18E-06	PRF
AX-399427267	4	60395776	60599459	60599529	7.24E-06	PRF
AX-399406221	4	60389795	60593478	60593548	8.09E-06	PRF
AX-399427636	4	60439904	60643531	60643600	8.11E-06	PRF
AX-391397334	4	59342799	59502015	59502082	1.03E-05	PRF
AX-399418571	4	59350770	59510080	59510150	1.03E-05	PRF
AX-391995141	4	59391254	59550168	59550238	1.14E-05	PRF
AX-391995289	4	59437092	59595963	59596033	1.14E-05	PRF
AX-399428424	4	60516652	60720182	60720252	1.51E-05	PRF
AX-399408850	4	60599450	60802878	60802948	1.62E-05	PRF
AX-399403252	4	57648391	57800629	57800699	1.68E-05	PRF
AX-391989691	4	57536809	57688046	57688116	1.86E-05	PRF

1 Positions of SNPs that were initially based on a private genome assembly.

2 Positions of SNPs that were based on ZJU1.0 assembly using NCBI BLAST.
