# Supplementary material for: Leveraging genome-wide association analyses with chip and imputed data emerges potential pleiotropic region for four duck growth traits
Source: Sci Rep. 2025 Jul 2;15:23625. doi: 10.1038/s41598-025-08852-z (PMC12223076; doi:10.1038/s41598-025-08852-z)
Supplement: Supplementary file 4 — Supplementary Material 4 [file 41598_2025_8852_MOESM4_ESM.pdf]

Supplementary Table S4. Common markers between GWAS analyses with medium density and imputed data.
Positional candidate genes are also given. Gene containing a marker are highlighted in bold.

**Marker**	**Chromosome**	**Position (bp) 1**	**Start of marker's alignment (bp) 2**	**End of marker's alignment (bp) 2**	**Trait**	**Positional candidate genes**
AX-406389417	4	57496977	57648845	57648915	ADG,BW,PRF, BDCOV	*LOC113843641,CCDC149,LOC119716791,SOD3,LOC 106017027,LOC106017022,LOC119716878,LOC113843504,DHX15, PPAR GC1A*
AX-391989691	4	57536809	57688046	57688116	ADG,BW,PRF	*CCDC149,LOC119716791,SOD3,LOC106017027,LOC 106017022,LOC119716878,LOC113843504,* ***DHX15*** *,PPARGC1A*
AX-399380444	4	57540987	57692215	57692285	ADG,BW	*CCDC149,LOC119716791,SOD3,LOC106017027,LOC 106017022,LOC119716878,LOC113843504,* ***DHX15*** *,PPARGC1A*

AX-399380966	4	57576167	57727323	57727393	ADG,BW,PRF,BDCOV	*LOC106017022,LOC119716878,LOC113843504,* ***DHX15*** *, PPARGC1A*
AX-391989849	4	57583530	57735769	57735839	ADG,BW,PRF,BDCOV	*LOC113843504,D HX15,* ***PPARGC1A***
AX-399381139	4	57590834	57743075	57743145	ADG,BW,PRF,BDCOV	*LOC113843504,D HX15,* ***PPARGC1A***
AX-413288581	4	57617544	57769805	57769875	ADG,BW,PRF,BDCOV	*DHX15,* ***PPARGC1A***
AX-399382231	4	57671217	57823432	57823502	ADG	***PPARGC1A***
AX-399404909	4	57744173	57895658	57895727	ADG,BW,PRF,BDCOV	***PPARGC1A***
AX-399384273	4	57770401	57921837	57921907	ADG,BW,PRF,BDCOV	***PPARGC1A***
AX-399385210	4	57818524	57969925	57969993	ADG,BW,PRF,BDCOV	***PPARGC1A***
AX-247869802	4	57859539	58010948	58011018	ADG,BW,PRF,BDCOV	***PPARGC1A*** *,LOC 106014335,LOC10 6014334*
AX-399407752	4	57869153	58020559	58020629	ADG,BW,PRF,BDCOV	***PPARGC1A*** *,LOC 106014335,LOC10 6014334*
AX-391395264	4	57874202	58025604	58025673	ADG,BW,PRF,BDCOV	***PPARGC1A*** *,LOC 106014335,LOC10 6014334*
AX-399386541	4	57883079	No significant similarity found by BLAST	No significant similarity found by BLAST	ADG,BW,PRF,BDCOV	

AX-399408144	4	57890046	58041452	58041522	ADG,BW,PRF,BDCOV	***PPARGC1A***,*LOC106014335,LOC106014334*
AX-399392532	4	58527810	58669666	58669736	ADG,BW,PRF,BDCOV	***KCNIP4***
AX-399393490	4	58693993	58836193	58836263	ADG,BW,PRF,BDCOV	***KCNIP4***
AX-399393735	4	58713747	58855924	58855994	ADG,BW,PRF,BDCOV	***KCNIP4***
AX-391396430	4	58736437	58878645	58878715	ADG,BW,PRF,BDCOV	***KCNIP4***
AX-391993165	4	58753852	58896057	58896127	ADG,BW,PRF,BDCOV	***KCNIP4***,*PACRGL*
AX-399415377	4	58758300	58900505	58900575	ADG,BW,PRF,BDCOV	***KCNIP4***,*PACRGL*
AX-399415535	4	58780463	58922661	58922731	ADG,BW,PRF,BDCOV	***KCNIP4***,*PACRGL,SLIT2*
AX-247856762	4	58809591	58951796	58951866	ADG, BW, PRF, BDCOV	***KCNIP4***,*PACRGL,SLIT2*
AX-391396574	4	58838915	58981046	58981116	ADG,BW,PRF,BDCOV	***KCNIP4***,*PACRGL,SLIT2*
AX-406389915	4	58840414	58982550	58982620	ADG,BW,PRF,BDCOV	*KCNIP4,* ***PACRGL***,*SLIT2*
AX-399394817	4	58884082	59026179	59026249	ADG,BW,PRF,BDCOV	*KCNIP4,PACRGL,* ***SLIT2***
AX-399396415	4	59227499	59386800	59386870	ADG,BW,PRF,BDCOV	*LOC113843643*
AX-399417739	4	59264682	59423996	59424066	ADG, BW, PRF, BDCOV	*LOC113843643*
AX-247857157	4	59375105	59534539	59534609	ADG, BW, PRF, BDCOV	*LOC113843643*

AX-391397463	4	59406999	59565894	59565963	ADG,BW,PRF,BDCOV	*LOC113843643*
AX-399419051	4	59410596	59569489	59569559	ADG, BW, PRF, BDCOV	*LOC113843643*
AX-391995514	4	59520360	59679121	59679191	ADG,BW,PRF,BDCOV	*LOC106014982*
AX-399420827	4	59663391	59822418	59822488	ADG, BW, PRF, BDCOV	*LOC106014982*
AX-399400325	4	59742341	59947561	59947631	ADG,BW,PRF,BDCOV	*LOC119716854,LCORL*
AX-399421434	4	59747809	59953029	59953099	ADG, BW, PRF, BDCOV	*LOC119716854,LCORL*
AX-247871298	4	59782113	59987248	59987318	ADG,BW,PRF,BDCOV	*LOC119716854,LCORL*
AX-391398482	4	60078280	60283368	60283438	ADG	*LAP3,CLRN2,QDPR,LOC106014980*
AX-399426907	4	60352376	60556065	60556131	ADG	***LDB2*** *,LOC119716757,LOC119716756,LOC119716899,LOC119716898*
AX-399406221	4	60389795	60593478	60593548	ADG,BW,PRF,BDCOV	***LDB2*** *,LOC119716757,LOC119716756,LOC119716899,LOC119716898,TAPT1*

AX-399427267	4	60395776	60599459	60599529	ADG, BW,PRF,BDCOV	***LDB2*** *,LOC119716757,LOC119716756,LOC119716899,LOC119716898,T APT1*
AX-399406282	4	60401337	60605036	60605106	ADG,BW,PRF, BDCOV	***LDB2*** *,LOC119716757,* ***LOC119716756*** *,LOC119716899,LOC119716898,TAPT1*
AX-399406303	4	60406158	60609856	60609926	ADG,BW,PRF, BDCOV	***LDB2*** *,LOC119716757,LOC119716756,LOC119716899,LOC119716898,TAPT1*
AX-399427492	4	60423459	60627102	60627172	ADG, BW,PRF,BDCOV	*LDB2,LOC119716757,LOC11971675 6,* **LOC119716899** *,LOC119716898,T APT1*
AX-399427636	4	60439904	60643531	60643600	ADG, BW,PRF,BDCOV	*LDB2,LOC119716757,LOC119716756,LOC119716899,* **LOC119716898** *,T APT1,PROM1*

AX-399406967	4	60475137	60678682	60678752	ADG	*LDB2,LOC119716757,LOC119716756,LOC119716899,LOC119716898,* ***TAPT1*** *,PROM1*
AX-399428424	4	60516652	60720182	60720252	ADG,BW,PRF,BDCOV	*LOC119716899,LOC119716898,TAPT1,PROM1,FGFBP2,LOC1197168 03*
AX-391998834	4	60555232	60758697	60758766	ADG	*TAPT1,* ***PROM1*** *,FGFBP2,LOC119716803,FGFBP1,BS T1*
AX-399408850	4	60599450	60802878	60802948	ADG,BW,PRF,BDCOV	*TAPT1,PROM1,FGFBP2,LOC119716803,FGFBP1,BST1,LOC110352211,FBXL5*
AX-247858317	4	60642080	60845604	60845674	ADG	*PROM1,FGFBP2,LOC119716803,F GFBP1,* ***BST1*** *,LOC110352211,FBXL5,CC2D2A*
AX-399411060	4	60711653	60915673	60915743	ADG,BW,BDCOV	*LOC119716803,FGFBP1,BST1,LOC110352211,* ***FBXL5*** *,CC2D2A,C1QT NF7*

AX-399411115	4	60720619	60924638	60924708	ADG,BW,BDCOV	*LOC119716803,FGFBP1,BST1,LOC110352211,* ***FBXL5*** *,CC2D2A,C1QT NF7*
AX-399416702	4	60729108	No significant similarity found by BLAST	No significant similarity found by BLAST	ADG,BW,BDCOV	-
AX-399432307	4	60738364	60942366	60942436	ADG,BW,BDCOV	*BST1,LOC110352 211,FBXL5,* ***CC2D 2A*** *,C1QTNF7*
AX-399432939	4	60803833	61007847	61007917	ADG,BW,PRF	*FBXL5,CC2D2A,C1QTNF7,LOC1197 16928,CPEB2*
AX-399412691	4	60839719	61043666	61043736	ADG,BW,PRF	*CC2D2A,* ***C1QTN F7*** *,LOC11971692 8,CPEB2*
AX-399413292	4	60866402	61070334	61070404	ADG,BW,PRF	*CC2D2A,C1QTNF 7,LOC119716928, CPEB2*
AX-399414599	4	60943298	61147146	61147216	ADG,BW,PRF	*C1QTNF7,LOC11 9716928,* ***CPEB2*** *, LOC119716930,LOC106016203,LOC119716873*
AX-399436070	4	60972754	61176572	61176642	ADG,BW,PRF	*LOC119716928,CPEB2,LOC119716 930,LOC10601620 3,LOC119716873*

AX-399436319	4	61001049	61204792	61204862	ADG,BW,PRF	*CPEB2,LOC119716930,LOC106016203,LOC119716873*
AX-247858741	4	61032304	61236036	61236106	ADG,BW,PRF	*CPEB2,LOC119716930,LOC106016203,LOC119716873*
AX-399437119	4	61085303	61289055	61289117	ADG,BW,PRF	*LOC106016203,LOC119716873,LOC119716923*
AX-247872616	4	61090701	61294438	61294508	ADG,BW,PRF	*LOC106016203,LOC119716873,LOC119716923*
