# Supplementary material for: Leveraging genome-wide association analyses with chip and imputed data emerges potential pleiotropic region for four duck growth traits
Source: Sci Rep. 2025 Jul 2;15:23625. doi: 10.1038/s41598-025-08852-z (PMC12223076; doi:10.1038/s41598-025-08852-z)
Supplement: Supplementary file 5 — Supplementary Material 5 [file 41598_2025_8852_MOESM5_ESM.pdf]

Supplementary Table S5. Descriptive statistics for the medium density and imputed data.

Description	Medium density data	Imputed data
**Pedigree**		
identities in pedigree	1785	13329
N of generations on Sire side	2 to 4	2 to 6
N of generations on Dam side	2 to 4	2 to 6
N of Sires	185	253
N of Sire_of_Sire	46	84
N of Dam_of_Sire	76	133
N of Dams	534	882
N of Sire_of_Dam	83	159
N of Dam_ofDam	184	343
**Data records**		
N. of data records	1445	13020
N.of males	339	6377
N. of females	1106	6643
N. of Hatch	20	23
Min records in hatch	29	30
Max records in Hatch	129	886
mean number of records per hatch	72	566
