# Supplementary figures and images for: Leveraging genome-wide association analyses with chip and imputed data emerges potential pleiotropic region for four duck growth traits
Source: Sci Rep. 2025 Jul 2;15:23625. doi: 10.1038/s41598-025-08852-z (PMC12223076; doi:10.1038/s41598-025-08852-z)

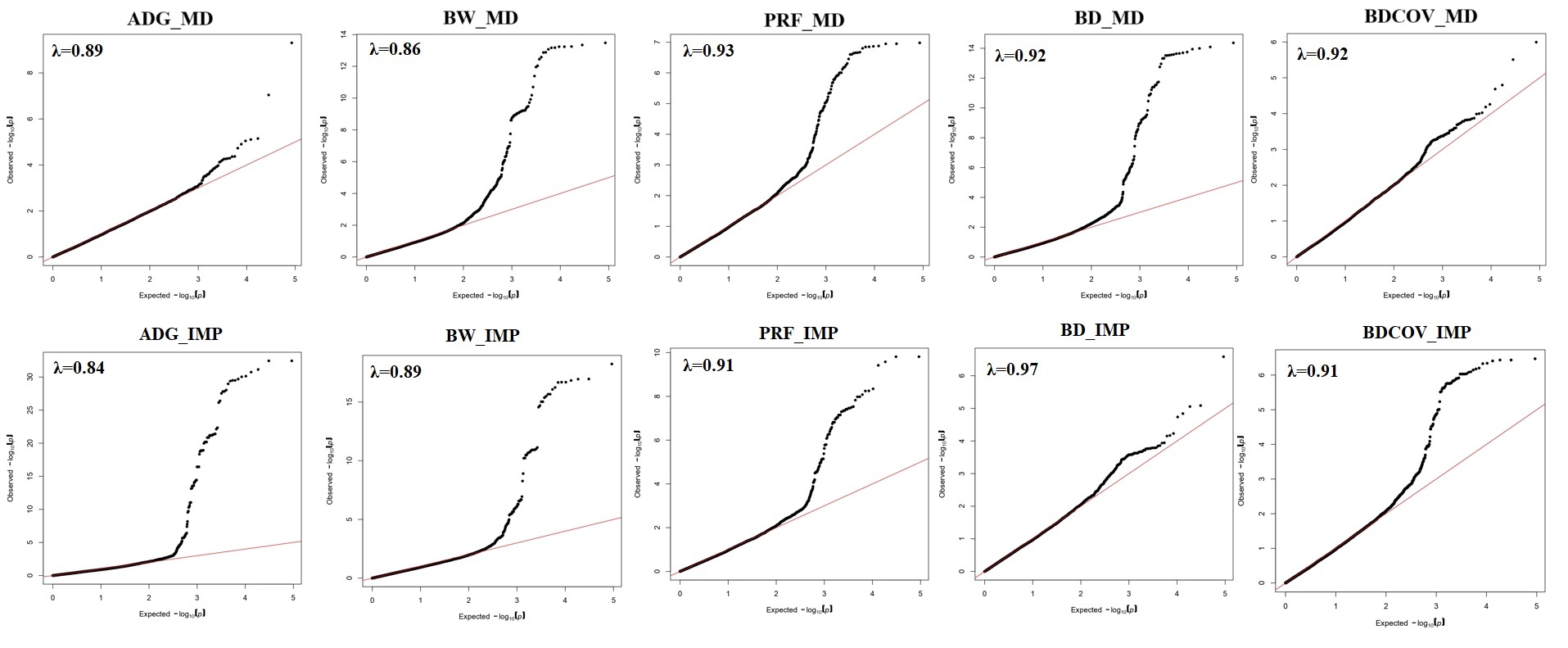

Supplement: Supplementary file 6 — Supplementary Material 6 [file 41598_2025_8852_MOESM6_ESM.jpg]

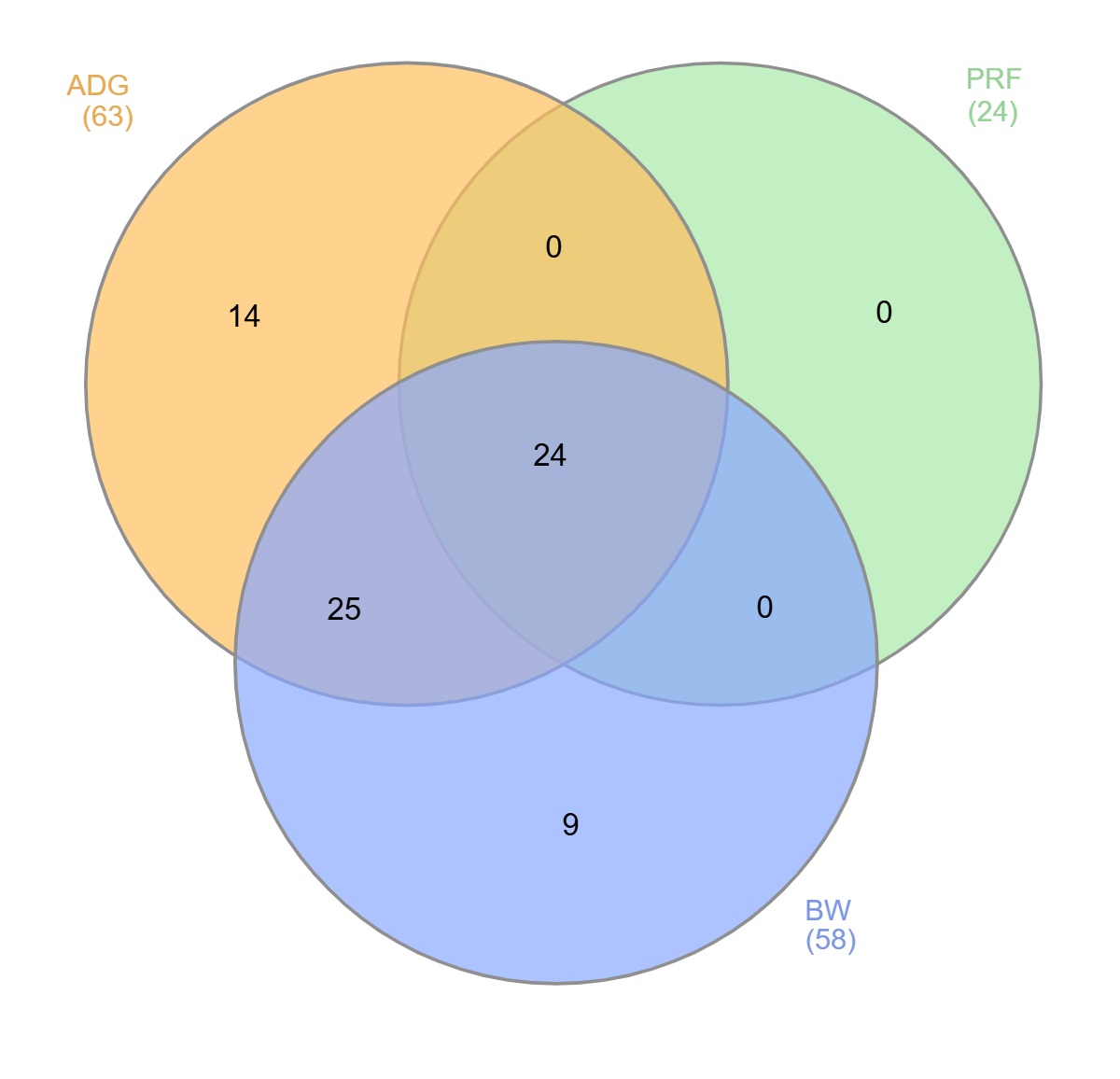

Supplement: Supplementary file 7 — Supplementary Material 7 [file 41598_2025_8852_MOESM7_ESM.jpg]

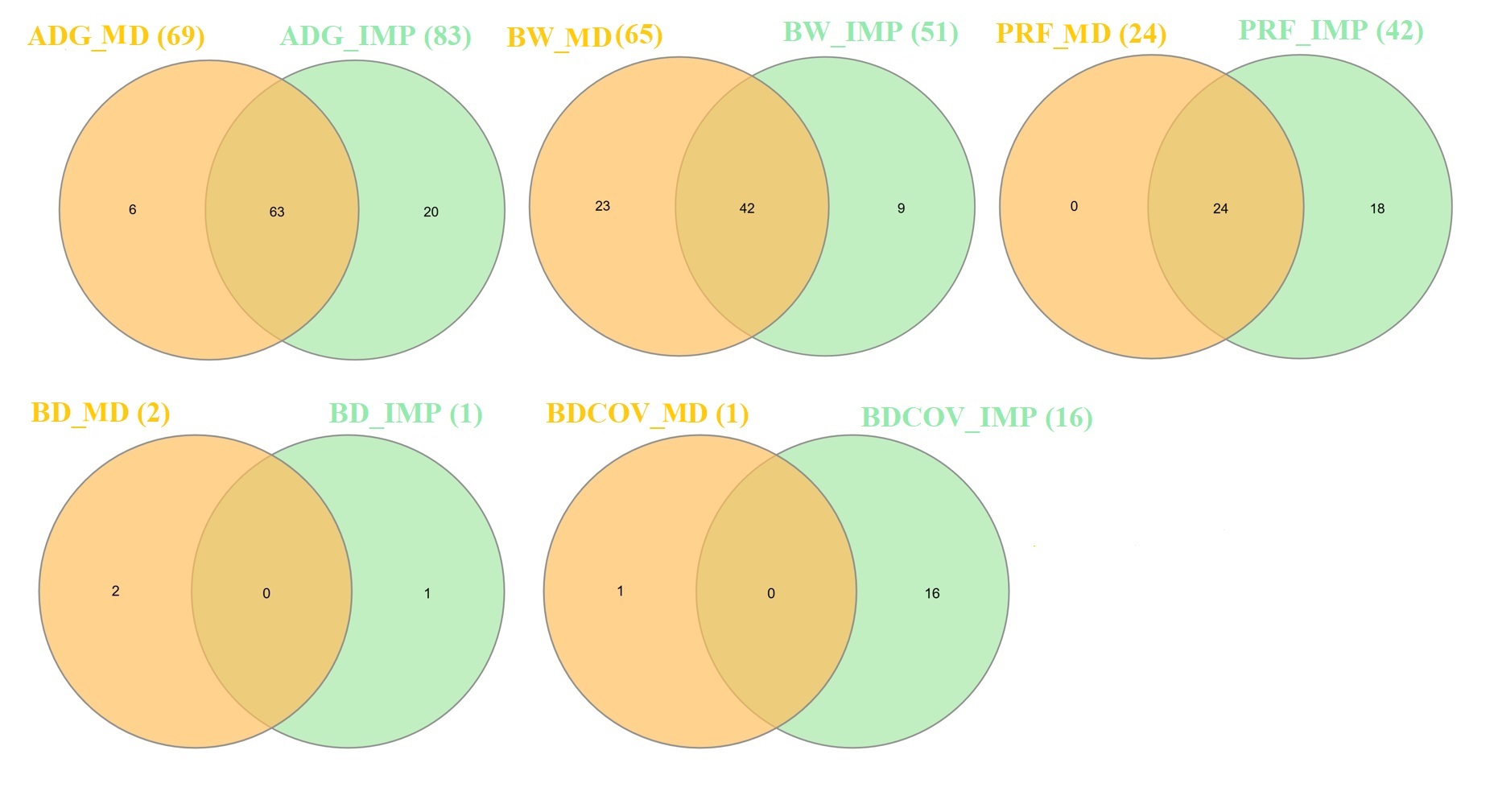

Supplement: Supplementary file 8 — Supplementary Material 8 [file 41598_2025_8852_MOESM8_ESM.jpg]

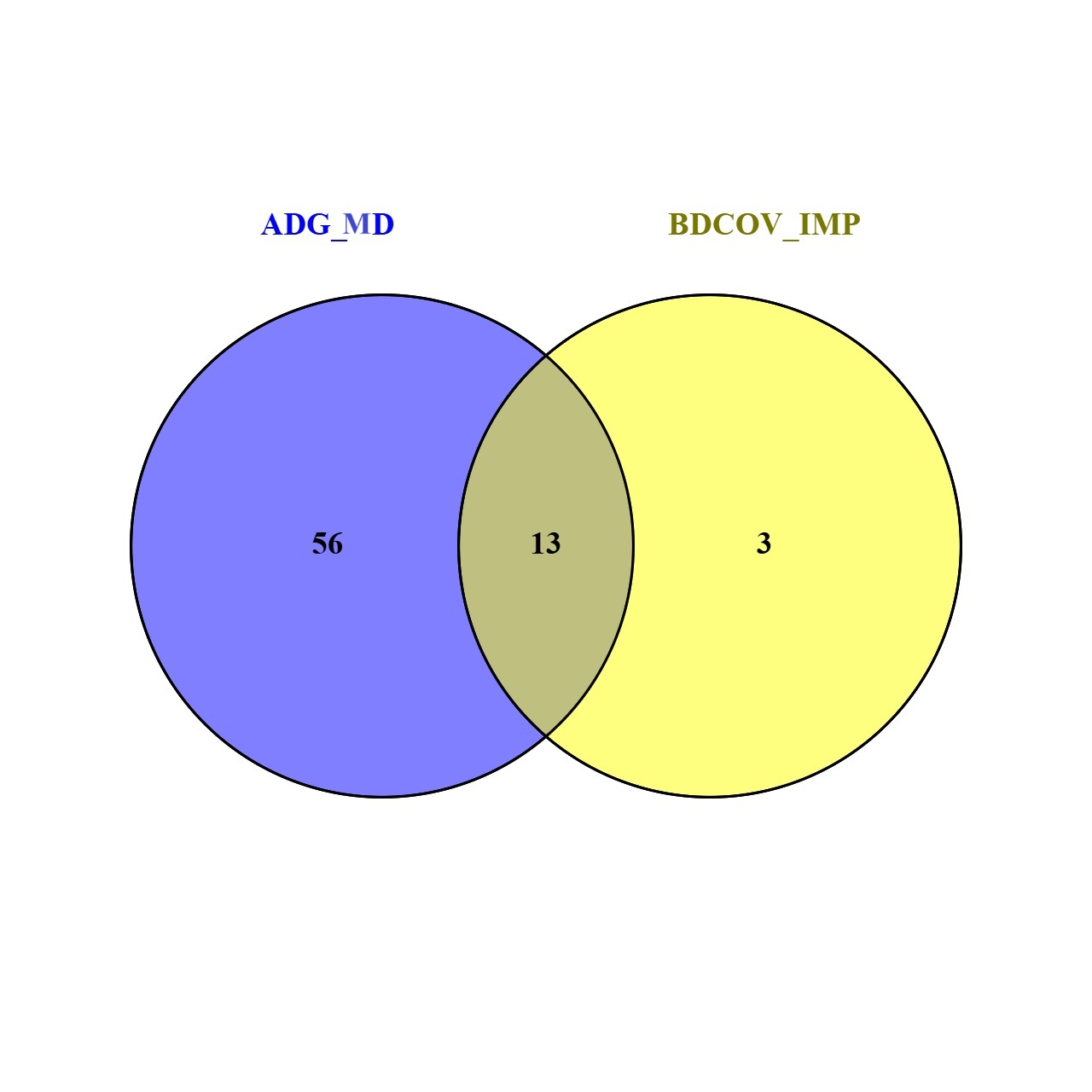

Supplement: Supplementary file 9 — Supplementary Material 9 [file 41598_2025_8852_MOESM9_ESM.jpg]

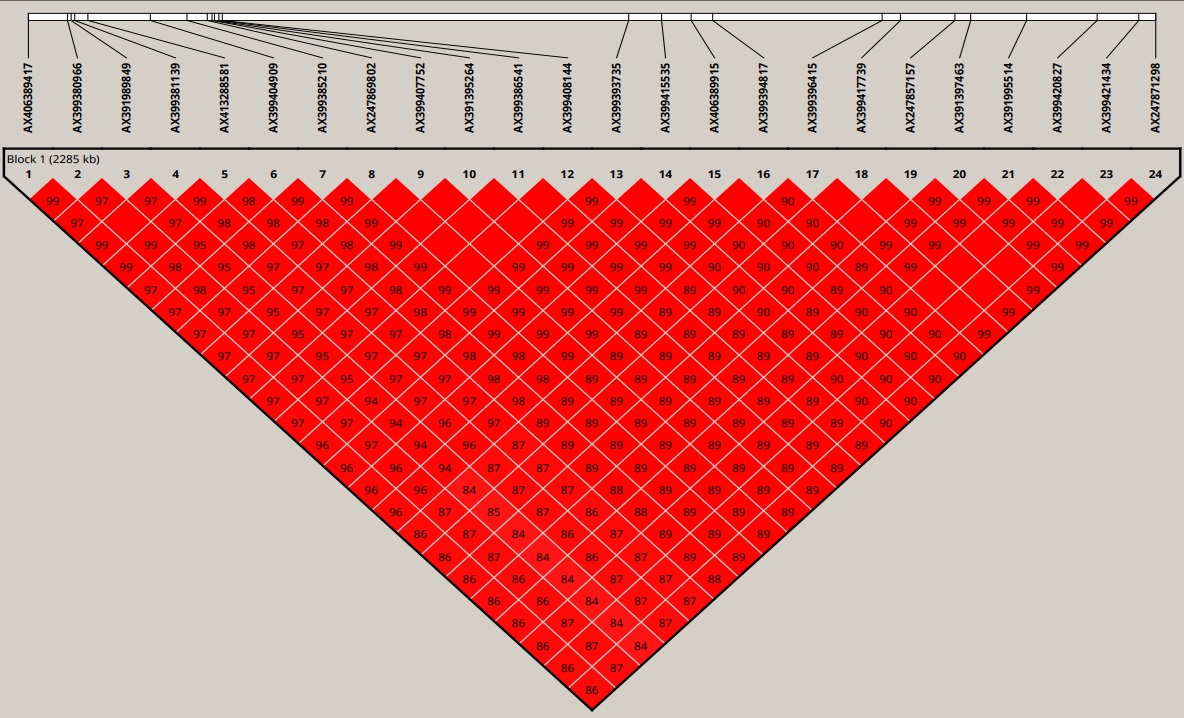

Supplement: Supplementary file 10 — Supplementary Material 10 [file 41598_2025_8852_MOESM10_ESM.jpg]
