# Supplementary material for: Leveraging genome-wide association analyses with chip and imputed data emerges potential pleiotropic region for four duck growth traits
Source: Sci Rep. 2025 Jul 2;15:23625. doi: 10.1038/s41598-025-08852-z (PMC12223076; doi:10.1038/s41598-025-08852-z)
Supplement: Supplementary file 11 — Supplementary Material 11 [file 41598_2025_8852_MOESM11_ESM.docx]

**Supplementary Figure 1**. Q-Q plots along with the genomic inflation factor (λ) estimates from all genomic analyses for MD and imputed data.

**Supplementary Figure 2.** Venn diagram showing the number of common significant SNPs between ADG, BW and PRF on chromosome 4 for the MD data.

**Supplementary Figure 3.** Venn diagrams showing the numbers of common significant SNPs between the MD and imputed data for the examined traits on chromosome 4.

**Supplementary Figure 4.** Venn diagram showing the number of common significant SNPs between ADG_MD and BDCOV_IMP.

**Supplementary Figure 5.** LD heatmap for the top 24 out of the 63 SNPs (chromosome 4) between MD and imputed genotypes.
